# A single dose recombinant AAV based CHIKV vaccine elicits robust and durable protective antibody responses in mice

**DOI:** 10.1371/journal.pntd.0012604

**Published:** 2024-11-04

**Authors:** Qin-Xuan Zhu, Ya-Nan Zhang, Hong-Qing Zhang, Chao Leng, Cheng-Lin Deng, Xin Wang, Jia-Jia Li, Xiang-Li Ye, Bo Zhang, Xiao-Dan Li

**Affiliations:** 1 School of Medical Technology and Translational Medicine, Hunan Normal University, Changsha, China; 2 Key Laboratory of Special Pathogens and Biosafety, Wuhan Institute of Virology, Center for Biosafety Mega-Science, Chinese Academy of Sciences, Wuhan, China; 3 University of Chinese Academy of Sciences, Beijing China; Colorado State University, UNITED STATES OF AMERICA

## Abstract

**Background:**

Chikungunya virus (CHIKV) is a mosquito-borne alphavirus that is responsible for Chikungunya fever, which is characterized by fever, rash, and debilitating polyarthralgia. Since its re-emergence in 2004, CHIKV has continued to spread to new regions and become a severe health threat to global public. Development of safe and single dose vaccines that provide durable protection is desirable to control the spread of virus. The recombinant adeno-associated virus (rAAV) vectors represent promising vaccine platform to provide prolonged protection with a single-dose immunization. In this study, we developed a rAAV capsid serotype 1 vector based CHIKV vaccine and evaluated its protection effect against CHIKV challenge.

**Methodology:**

The recombinant AAV1 encoding the full-length structural proteins of CHIKV (named as rAAV1-CHIKV-SP) was generated *in vitro* by transfecting the plasmids of AAV helper-free system into HEK-293T cells. The safety and immunogenicity of rAAV1-CHIKV-SP were tested in 4-week-old C57BL/6 mice. The antibody responses of the mice receiving prime-boost or single-dose immunization of the vaccine were determined by ELISA and plaque reduction neutralizing test. The immunized mice were challenged with CHIKV to evaluate the protection effect of the vaccine.

**Conclusions:**

The rAAV1-CHIKV-SP showed remarkable safety and immunogenicity in C57BL/6 mice. A single dose intramuscular injection of rAAV1-CHIKV-SP elicited high level and long-lasting antibody responses, and conferred complete protection against a heterologous CHIKV strain challenge. These results suggest rAAV1-CHIKV-SP represents a promising vaccine candidate against different CHIKV clades with a simplified immunization strategy.

## Author summary

Recently, a live attenuated CHIKV was approved as the first vaccine to combat the virus infection. Despite the general safety of this vaccine, several severe chikungunya-like adverse reactions have been reported, and the long-term safety should be assessed after marketing, highlighting the urgent need to develop more safe and effective vaccines against CHIVK. The rAAV vectored vaccines against several viruses such as SARS-CoV-2, HIV, HCV and Nipah virus have been developed. Here, we constructed a rAAV capsid serotype 1 based CHIKV vaccine that encoded the full-length structural proteins of CHIKV (rAAV1-CHIKV-SP), and evaluated the safety, immunogenicity and protection effect of the vaccine. The results demonstrated that a single dose immunization of the vaccine induced high level and long-lasting CHIKV-specific antibodies and neutralizing antibodies, and completely protected against CHIKV challenge in C57BL/6 mice. Our study suggested that the rAAV1-CHIKV-SP represents a promising vaccine candidate to prevent CHIKV infection.

## Introduction

Chikungunya virus (CHIKV) is a re-emerging mosquito-borne virus belonging to the *Alphavirus* genus from the *Togaviridae* family. Due to the wide geographic distribution of the mosquito vectors and the viral adaptive evolution to its vectors and hosts, CHIKV has spread to more than 100 countries in Africa, Asia, Europe, and the Americas, and caused several large-scale outbreaks worldwide since its re-emergence in 2004 in Kenya [1]. Human CHIKV infection results in a self-limiting disease known as Chikungunya fever (CHIKF) which is characterized by abrupt fever, headache, rash, myalgia and polyarthralgia. The polyarthralgia often transitions into chronic, incapacitating arthritis that lasts weeks to months or even years, severely affecting the life quality of the patients and imposing a huge financial burden to the families and society. In the newborns, the elderly, and individuals with pre-existing medical conditions, CHIKF may progress to severe illness that can increase the risk of death [2]. In addition, mother-to-fetus vertical CHIKV transmission has been reported to cause pregnancy termination or neonatal sequelae including encephalitis, neurocognitive delays, or microcephaly [3, 4]. Despite posing these serious threats to human health, there is no specific antiviral treatment against CHIKV infection. Vaccines provide the most effective and cost-efficient means to combat infectious diseases. Recently, a live attenuated vaccine, named as Ixchiq (VLA1553), was approved as the first CHIKV vaccine by U.S. Food and Drug Administration (FDA). However, this vaccine may cause symptoms that are similar to the wild type pathogenic CHIKV infection [5], and development of more safe and effective vaccines remains essential.

CHIKV is an enveloped virus with a single-stranded, positive-sense RNA genome encoding four non-structural proteins (nsP1~nsP4) that mediate RNA transcription and replication, and five structural proteins (capsid, E3, E2, 6K/TF and E1) which constitute the virus particle. The capsid protein associates with genome RNA, forming the central nucleocapsid of the virion. The two transmembrane glycoproteins E2 and E1 form heterodimers that are exposed on the virion surface as viral spike. Both E2 and E1 are essential for virus infection, the E2 interacts with host receptors and facilitates viral binding and attachment, and E1 mediates the fusion of the virus and endosome membranes after viral entry. It has been demonstrated that the neutralizing antibodies of alphaviruses mainly target to the E2 protein [6]. Therefore, the E2 glycoprotein or full-length structural proteins are the preferred antigen for CHIKV vaccine design. Currently, various CHIKV vaccines are under different stages of clinical trial evaluation, including a virus-like particle (VLP) vaccine at Ph3, a whole-virus inactivated vaccine at Ph2/3, a measles-vectored vaccine at Ph2, and mRNA-based and adenovirus vectored vaccines at Ph1 respectively. Among them, the two viral vector (measles virus and adenovirus) -based vaccines expressing CHIKV structural proteins represent promising vaccine candidates with good safety and immunogenicity that a single immunization of which could provide solid protection [7, 8]. Both measles virus and adenovirus vectors are widely used in vaccine development due to their advantages of high efficiency of gene delivery and expression in several cell types and induction of robust immune responses. However, the innate immunogenicity of the viral vector proteins may interfere with the antigen expression, and the existence of pre-existing immune responses to these common viral vectors in the host may also reduce the antigenicity of the vectored antigen, thereby affecting the vaccine efficacy.

The recombinant adeno-associated virus (rAAV) vector is derived from the non-pathogenic wild type AAV in which all of the viral coding regions are replaced by the gene of interest (GOI). Comparing to other viral vectors, the rAAV has several unique advantages. First, rAAV is a replication-deficient entity that only has the capability of infecting cells and delivering the GOI-containing DNA into the nucleus. The delivered DNA can form circular concatemers and persistently exist as episomes in the cell nucleus, resulting in long-term and stable expression of the GOI [9]. Since the rAAV DNA lacks viral coding sequences, there is no viral protein expression in the transduced cells, avoiding the potential safety concerns of the viral proteins and also reducing the innate immunogenicity of the viral vector. Accordingly, the rAAV vector has been demonstrated to have lower immunogenic profile comparing to the adenovirus and lentivirus vectors [10,11]. In addition, several natural AAV serotypes with different tissue or cell type tropism have been isolated, enriching the choice of the vectors for distinct therapeutic purposes. These advantages make rAAVs one of the most promising gene therapy platforms for decades. To date, a few rAAV-based gene therapies for different genetic diseases have been approved and more than 100 rAAV-based medications are under clinical trial evaluation. In addition to its wide application in the field of gene therapy, the use of rAAV as vaccine vectors has also been explored. The rAAV vectored vaccines against HSV, HIV, HCV, HSV, HPV, SARS-CoV-2, influenza, Ebola and Nipah virus have been demonstrated to elicit strong immune responses and provide efficient protection against viral challenges [12–15], indicating the potential of rAAV as vaccine platform.

In this study, we developed a safe and effective CHIKV vaccine based on the rAAV1 vector which is the most efficient serotype for skeletal muscles transduction and has been approved for use in gene therapy [16]. We constructed the rAAV1 encoding the full-length structural proteins of CHIKV (rAAV1-CHIKV-SP), and evaluated its safety, immunogenicity, and protection effect against CHIKV in the C57BL/6 mouse model. The rAAV1-CHIKV-SP exhibited good safety profile, and a single intramuscular injection could induce robust and long-term immune responses, and provide complete protection against CHIKV challenge. These results suggest that the rAAV encoding CHIKV structural proteins can be served as an alternative vaccine candidate for CHIKV vaccine development.

## Materials and methods

### Ethics statement

The C57BL/6 mice were provided by the Animal Center of Wuhan Institute of Virology, Chinese Academy of Sciences. All the mice were cared in accordance with the recommendations of National Institutes of Health Guidelines for the Care and Use of Experimental Animals. Virus challenges of the mice were conducted in an animal biosafety level 3 (ABSL-3) facility at Wuhan Institute of Virology under a protocol approved by the Laboratory Animal Ethics Committee of Wuhan Institute of Virology, Chinese Academy of Sciences (Approval number: WIVA26202304).

### Cells, viruses, and antibodies

The HEK-293T cells, Vero cells and BHK-21 cells were cultured in Dulbecco’s modified Eagle’s medium (DMEM, Invitrogen, Germany) containing 10% fetal bovine serum (FBS), 100 U/mL of penicillin, and 100 mg/mL of streptomycin at 37°C with 5% CO_2_. The wild-type (WT) CHIKV virus was recovered from the infectious cDNA clone of an Asian strain (GenBank accession No. KC488650) in BHK-21 cells as we described previously [17]. The CHIKV East/Central/South/Africa (ECSA) strain was propagated from the virus stock which was isolated from a Pakistan patient during the virus outbreak in 2016–2017 [18] on Vero cells. The polyclonal antiserum against CHIKV E2 protein was produced by immunizing the mouse with the recombinant E2 protein. The fluorescein isothiocyanate (FITC)-conjugated goat anti-mouse IgG antibody, and horseradish peroxidase (HRP)-conjugated goat anti-mouse IgG, IgG1, and IgG2c antibodies were purchased from Proteintech (China).

### Plasmid construction

**Fig 1:**
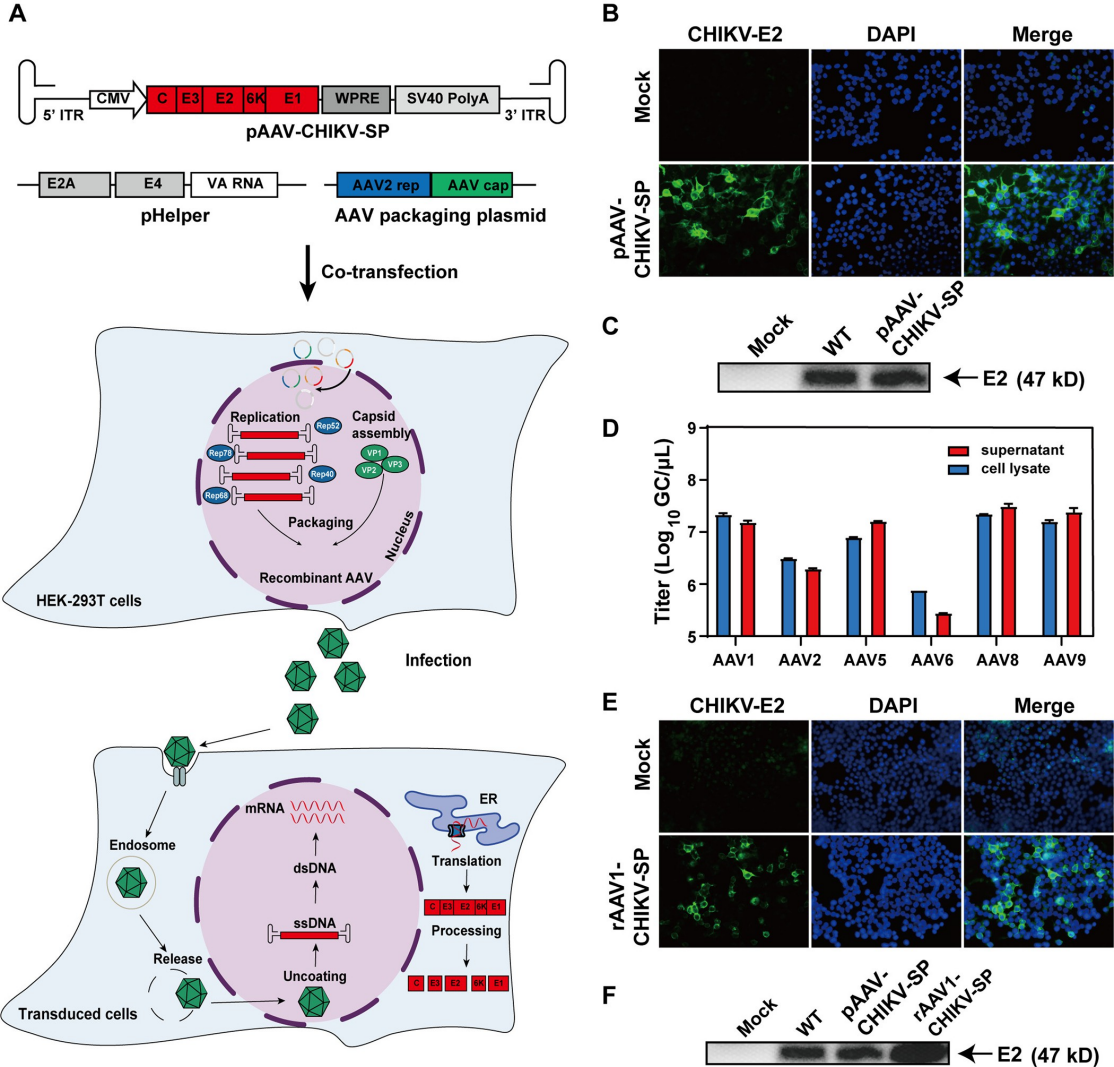
Construction and characterization of rAAV-CHIKV-SP. (A) The schematic representation of the recombinant genome and the processes of production and transduction of rAAV-CHIKV-SP. (B and C) The expression of CHIKV-E2 protein in pAAV-CHIKV-SP transfected HEK-293T cells at 48 hpt analyzed by immunostaining IFA (B) and western blot assay (C), respectively. (D) The genome titers of rAAV-CHIKV-SP produced by different AAV serotypes determined by qPCR. (E and F) The expression of CHIKV-E2 protein in HEK-293T cells infected with rAAV-CHIKV-SP at an MOI of 10^5^ detected by IFA (E) and western blot (F), respectively, at 72 hpi.

To construct the AAV vector encoding the CHIKV full-length structural proteins, the fragment containing the Capsid-E3-E2-6K-E1 genes was amplified using the WT CHIKV infectious clone as template, and cloned into the AAV expression vector containing a CMV promoter, a woodchuck hepatitis virus posttranscriptional regulatory element (WPRE), a SV40 polyA signal, and the AAV2 inverted terminal repeats (ITRs) at BamH I and Spe I restriction sites (Fig 1A). The constructed plasmid was named as pAAV-CHIKV-SP, and was verified by DNA sequencing.

### Production of the recombinant AAVs

The recombinant AAVs were generated by transfecting the three plasmids of the AAV helper-free system which are the GOI expression plamid (pAAV-CHIKV-SP or pAAV-eGFP), the AAV packaging-plasmid (providing the AAV2 *rep* gene and the *cap* gene of AAV1 or other AAV serotypes), and pHelper (providing the adenovirus E4, E2A, and VA RNA genes) into HEK-293T cells (providing the adenovirus E1A gene). Briefly, equimolar of the three plasmids with a total amount of 16 μg were co-transfected into HEK-293T cells which were maintained in a 10-cm culture dish and reached 70–80% confluence using the polyethyleneimine (PEI) reagent (Polysciences, Cat #24765–1) with a PEI/DNA ratio of 3:1 (v/w). At 72 hours post transfection (hpt), the supernatants were harvested, and the cells were scraped and resuspended in 1 mL PBS followed with three freeze-thaw cycles and centrifugation to clear the debris. The cell lysates and supernatants were incubated with 8% PEG8000 containing 0.5M NaCl at 4°C overnight, following centrifuging at 10,200 g for 1 hour at 4°C. The pellets were resuspended in PBS, aliquoted and frozen at -80°C. For titration of the rAAVs, 2 μL rAAVs stock was incubated with DNase at 37°C for 1 hour following being heated at 100°C for 10 minutes. The treated rAAVs were 1:1000 and 1:10,000 diluted in ddH_2_O, and genome titers (genome copies [GC] per milliliter) were determined by real-time quantitative PCR (qPCR) using the primers targeting to the ITR region of the AAV2 [19] according to the standard curve which is generated from the pAAV-CHIKV-SP plasmid.

### Indirect immunofluorescence assay (IFA)

HEK-293T cells seeded in six-well plates containing coverslips were transfected with pAAV-CHIKV-SP plasmid or infected with rAAV. At the indicated time points, the coverslips were collected, washed with PBS, and fixed with cold (-20°C) 5% acetic acid in acetone for 15 min at room temperature. After washing with PBS for three times, the fixed cells were incubated with the mouse anti-CHIKV E2 protein polyclonal antibody (1:250 dilution in PBS) at room temperature for 1 hour, followed by three times of wash with PBS and incubation with goat anti-mouse IgG antibodies conjugated with FITC (1:125 dilution in PBS) for another 1 hour. The nuclei were stained with DAPI (1 μg/mL in PBS) for 5 min, and the coverslips were mounted with 90% glycerol and examined under a fluorescence microscope (NIKON, Japan). The fluorescent images were taken at 400× magnification.

### Western blotting

HEK-293T cells were seeded into six-well plates and transfected with pAAV-CHIKV-SP plasmid or infected with the rAAV. At the indicated time points, the transfected or infected cells were lysed with 200 μL RIPA lysis buffer (Beyotime, China) on ice for 15 min. The cell lysates were heated at 95°C for 10 min, and subsequently separated by 10% SDS-PAGE and then electro-transferred onto a polyvinylidene fluoride (PVDF) membrane (Millipore). The blots were blocked with 5% skim milk in TBST (50 mM Tris-HCl, 150 mM NaCl, 0.1% Tween 20, pH 7.4) for 1 hour at room temperature. The blocked membranes were then incubated with the mouse anti-CHIKV E2 protein polyclonal antibody (1:2000 in dilution with TBST) at room temperature for 1 hour, followed by three times of wash with TBST and incubation with the HRP-conjugated goat anti-mouse antibody (1:2500 in dilution with TBST) at room temperature for 1 hour. The protein bands were visualized with a chemiluminescent HRP-conjugated antibody detection reagent (Millipore, USA) and a chemiluminescence system (ChemiDoc, Bio-Rad) according to the manufacturer’s protocols.

### Mouse experiments

For immunization and challenge experiments, 4-week-old female C57BL/6 mice (n = 5) were immunized with 5×10^10^ GC of rAAV1-CHIKV-SP or rAAV1-eGFP or same volume of PBS through intramuscular injection (IM) in the tibialis anterior muscle of the hind leg, and boosted with the same method on day 21. The weight change and health condition were recorded daily after immunization, and serum samples were collected on day 14 and 28 after the first immunization for determination of the titers of CHIKV-specific IgG antibodies and neutralizing antibodies. After 38 days of the first immunization, all the mice were challenged with 2.5×10^5^ PFU of CHIKV-ECSA subcutaneously (s.c.) in the ventral/lateral side of the hind foot. Footpad swelling was assessed by measuring the height and width of the peri-metatarsal area of the hind foot using Kincrome digital vernier calipers for five days, and viremia of the sera collected on 1, 2, and 3 days after challenge was determined by plaque assay. To further assess the protection effect, a single-dose of 5×10^8^, 5×10^9^, 5×10^10^ GC of rAAV1-CHIKV-SP were intramuscularly injected to the mice (n = 5), and the titers of CHIKV-E2 specific antibodies and neutralizing antibodies were examined on day 14 and 28, respectively. The mice were challenged with 2.5×10^5^ PFU of CHIKV-ECSA, the footpad swelling and viremia were examined after challenging. One group of the 5×10^10^ GC rAAV1-CHIKV-SP single injected mice were not challenged, and were maintained to 12 weeks after the vaccination, and the titers of CHIKV-E2 specific antibodies and neutralizing antibodies from the sera were detected on week 2, 4, 6, 8, 10 and 12, respectively.

### Enzyme linked immunosorbent assay (ELISA)

Indirect ELISA was used to detect the titers of CHIKV-specific IgG antibodies from the serum samples collected from the immunized mice. The 96-well microtiter plates were coated with inactivated WT CHIKV at 4°C overnight, and blocked with 5% skim milk for 1 hour at room temperature. The serially diluted sera were added to each well and incubated for 2 hours at 37°C. After washing with PBS for three times, the plates were incubated with HRP-conjugated goat anti-mouse IgG, IgG1, or IgG2c antibodies for 1 hours at 37°C. The 3,3′,5,5′-tetramethylbenzidine (TMB) substrate was added to the plates, following the addition of 1 M H_2_SO_4_ to stop the reaction. The absorbance at 450 nm was read by a multimode microplate reader (Varioskan Flash, Thermo Fisher).

### Plaque reduction neutralization test (PRNT)

The neutralizing activity of the serum samples from the immunized mice was analyzed by plaque reduction neutralization test (PRNT) as described previously [20]. Briefly, serial two-fold dilutions of the heat-inactivated serum samples were incubated with approximately 25 PFU of WT CHIKV at 37°C for 1 hour, and the viral titers of the mixtures were examined by plaque assay as described previously [20]. The neutralizing antibody titer was determined based on the highest serum dilution reducing 50% viral plaques compared with the WT virus control.

### Statistical analyses

All data were analyzed using GraphPad Prism 8.0.2 software and presented as means ± standard deviations (SD). Student’s *t* test was used to determine the statistical difference between two groups, and one-way ANOVA or two-way ANOVA tests were utilized for statistical analyses among multiple groups.

## Results

### Design, production, and identification of the recombinant AAV encoding the structural proteins of CHIKV

As one of the leading platforms for gene therapy, the recombinant AAV vectors have robust ability of *in vivo* gene delivery into a variety of tissues and cell types. However, it has been reported that the packaging capacity for the GOI of the AAV vector is restricted to ~3.3 kb [12], whereas the encoding sequence of the full-length structural polyprotein (Capsid-E3-E2-6K-E1) of CHIKV is 3.7 kb. To verify whether the rAAV vector can express and transfer the CHIKV structural polyprotein gene, a pAAV-CHIKV-SP plasmid, in which the Capsid-E3-E2-6K-E1 encoding sequence was inserted between the two AAV2 ITRs sequences and under the control of a CMV promoter (Fig 1A), was first constructed, and transfected into HEK-293T cells followed by IFA and Western blot assays using the CHIKV E2-specific antibody as primary antibody. The CHIKV E2 protein was detected in the pAAV-CHIKV-SP transfected cells but not in the non-transfected control cells (Fig 1B and 1C), and the size of E2 protein in the transfected cells was consistent with that from the WT-CHIKV infected cells (Fig 1C). We next compared the packaging efficiency of the different AAV serotype capsids for the structural polyprotein gene cargo. Equimolecular of pAAV-CHIKV-SP, the AAV packaging plasmids providing the AAV2 *rep* gene and varied AAV *cap* gene, and the pHelper plasmids were co-transfected into HEK-293T cells, and the supernatants and cell lysates containing the rAAV carrying the CHIKV structural polyprotein gene (rAAV-CHIKV-SP) were harvested at 72 hpt and titrated by real-time qPCR. As shown in Fig 1D, AAV1, AAV5, AAV8, and AAV9 packaging plasmids generated comparable amounts of rAAV-CHIKV-SP (with genome titers of higher than 10^7^ GC/μL) in both the supernatants and the cell lysates, whereas the AAV2 and AAV6 packaging plasmids showed much lower genome titers. Considering that rAAV1 has good transduction efficiency in mouse skeletal muscles [21], and it has been approved as the first viral vector for gene therapy, we selected rAAV1-CHIKV-SP for the subsequent trials. To test its transduction capacity, HEK-293T cells were infected with rAAV1-CHIKV-SP at an MOI of 10^5^, and the expression of CHIKV E2 protein was analyzed by IFA (Fig 1E) and Western blot assay (Fig 1F). The results showed that CHIKV E2 protein was efficiently expressed in the rAAV1-CHIKV-SP infected cells.

### Safety and immunogenicity of the rAAV-CHIKV-SP in C57BL/6 mice

**Fig 2:**
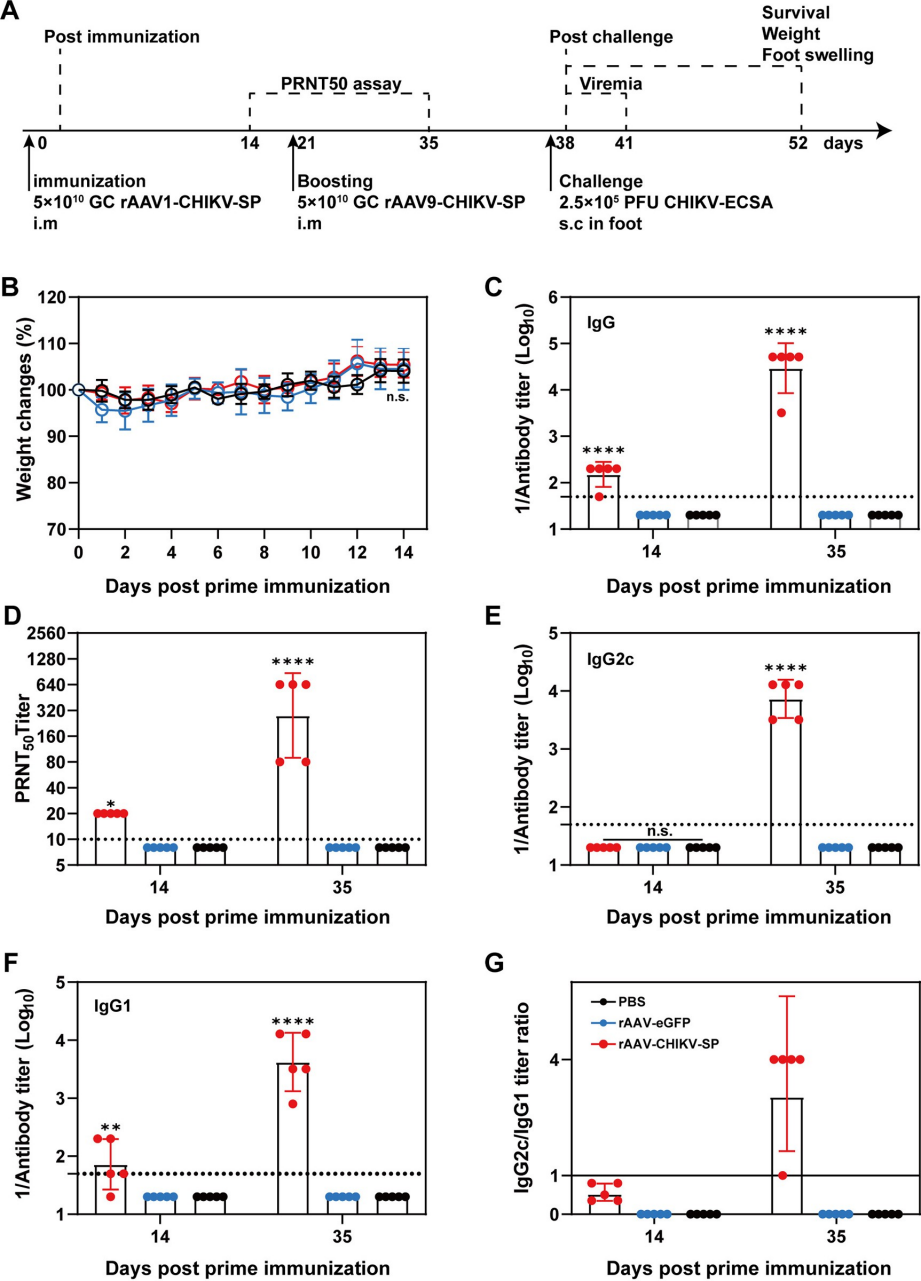
Antibody responses of C57BL/6 mice receiving prime-boost immunization of rAAV-CHIKV-SP. (A) Experimental schedule. Four-to-six weeks old C57BL/6 female mice were IM injected with 5 × 10^10^ GC of rAAV1-CHIKV-SP, followed by boosting with the same dose of rAAV9-CHIKV-SP on day 21. Mice given 5 × 10^10^ GC rAAV1-eGFP were used as empty vector control and mice given PBS were set as mock. The serum samples from each mouse harvested on day 14 and 35 were subjected to ELISA and PRNT assays. The mice were challenged with 2.5×10^5^ PFU CHIKV ECSA strain virus through s.c. injection in feet on day 38, viremia was determined, and the symptoms post-challenge were recorded at the indicated times post challenge. (B) Body weight change of the immunized mice compared with the PBS control group within 2 weeks after the primary immunization. (C and D) The titers of CHIKV-specific antibodies (C) and neutralizing antibodies (D) from the serum samples determined by ELISA and PRNT, respectively. (E to G) IgG subtype analysis by ELISA. The serum samples from each group were subjected to ELISA assay to measure the levels of IgG2c (E) and IgG1 (F), and IgG2c/IgG1 ratios (G) were calculated and presented. The dashed lines in panels B, C, D, and E represent the limits of detection. Data were represented as the mean ± standard deviation (n = 5 in each group). * *p* < 0.05, **p < 0.01, **** *p* < 0.0001. n.s., no statistical difference.

To assess the safety and immunogenicity of the rAAV vectored vaccine, groups of C57BL/6 mice were intramuscularly injected with 5×10^10^ GC of rAAV1-CHIKV-SP, rAAV1-eGFP or PBS (Fig 2A). The body weight and health condition of each mouse were recorded daily after the first injection for 14 days. We found that there was no significant difference in the body weight changes between the rAAV1-CHIKV-SP, rAAV1-eGFP and PBS injected mice (Fig 2B), and all the mice survived and demonstrated no signs of disease, indicating the safety of rAAV in C56BL/6 mice. The serum of each mouse was collected two weeks after the immunization and subjected to an indirect ELISA using the inactivated CHIKV virus as the immobilized antigen to determine the levels of CHIKV-specific antibodies. As shown in Fig 2C, the CHIKV-specific antibodies were detectable in the rAAV1-CHIKV-SP immunized mice but not in the rAAV1-eGFP and PBS injected mice, whereas the antibody titers were relatively low (1:200), suggesting a boost immunization might be required to augment the immune responses. To avoid the potential interference of the neutralizing antibody against AAV1 capsid which would possibly be induced after the first injection, we used rAAV9-CHIKV-SP (Fig 1D), which was packaged by a heterologous AAV9 serotype that has tropism towards skeletal muscles [22], as the second booster. The rAAV1-CHIKV-SP immunized mice were injected with 5×10^10^ GC of rAAV9-CHIKV-SP on day 21 post the first injection (Fig 2A). Two weeks after the boost immunization, the titers of CHIKV-specific antibodies significantly increased and reached 1:51,200 (Fig 2C). The production of neutralizing antibodies against CHIKV is crucial in the control of CHIKV infection [23]. To test whether the antibodies have neutralizing activity, the serum of each mouse after the first and second immunization was subjected to a plaque reduction neutralization test (PRNT) using the wild type CHIKV virus on BHK-21 cells. As shown in Fig 2D, consistent with the CHIKV-specific antibodies, the neutralizing antibodies were detectable in the mice vaccinated with rAAV-CHIKV-SP but not in the rAAV1-eGFP and PBS inoculated mice, and the 50% reduction in the number of virus plaques values (PRNT_50_) of the neutralizing antibodies increased from 1:20 after the first immunization to 1:320 after the booster vaccination. To further evaluate the quality of the induced antibodies, we had analyzed the IgG subtypes IgG2c and IgG1 which represents Th1 and Th2-type responses respectively. We found that the IgG1 rather than IgG2c was induced after the primary vaccination (Fig 2E and 2F). Both IgG1 and IgG2c were significantly elicited after the booster immunization, but the titer of IgG2c (1:12,800) was higher than that of IgG1 (1:3200), resulting a IgG2c/IgG1 ratio over 1 (Fig 2G), which is indicative of a Th1-biased immune response.

### The prime-boost immunization of rAAV-CHIKV-SP protected the C57BL/6 mice from CHIKV infection

**Fig 3:**
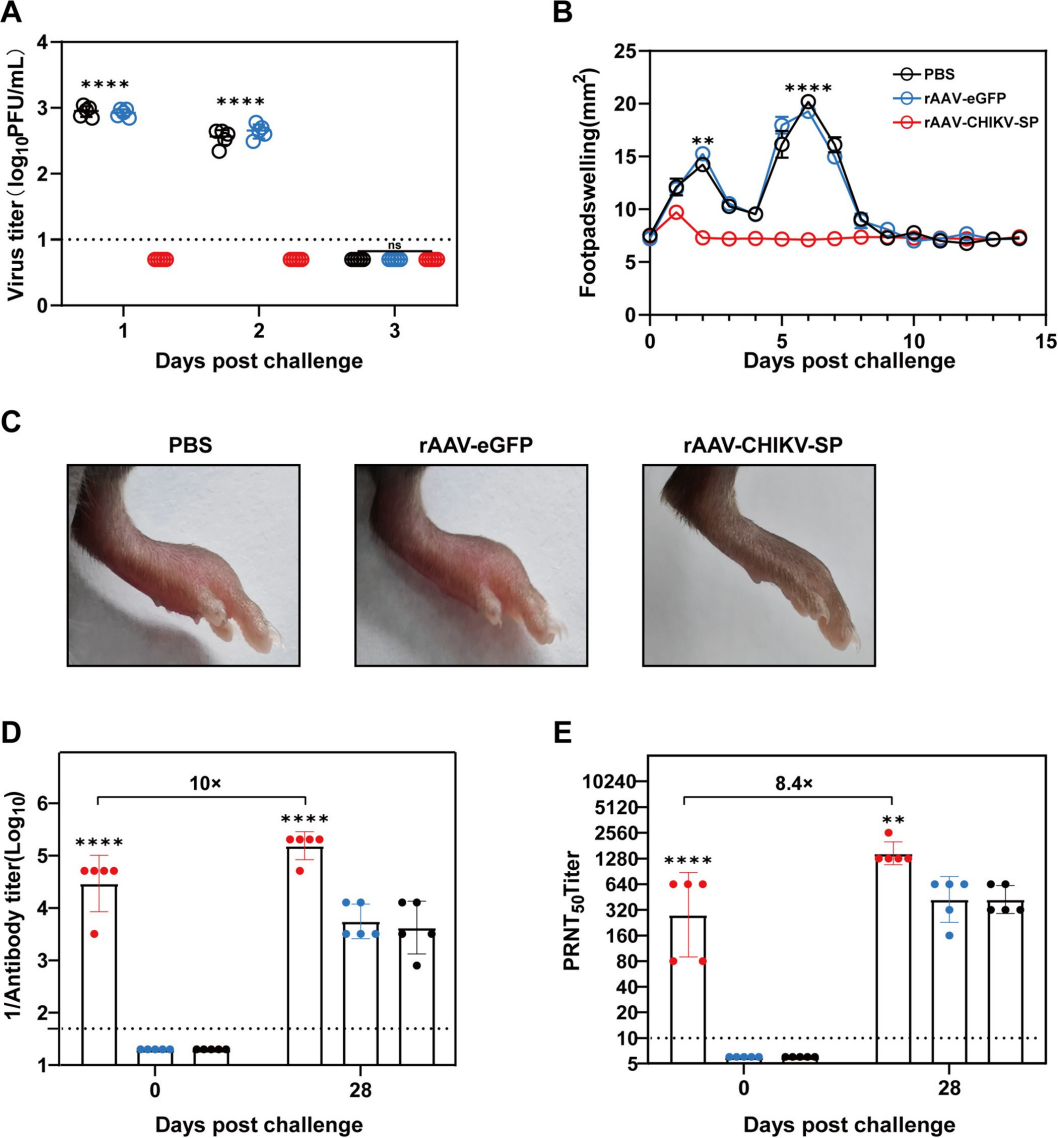
Prime-boost immunization of rAAV-CHIKV-SP protects C57BL/6 mice from CHIKV challenge. (A) Viremia post-challenge. Viremia of each mouse from day 1 to day 3 post-challenge was measured by plaque assay. (B) Footpad swelling examination after 14 days of challenge. (C) Representative images of the injected feet of the mice from each group on day 5 post-challenge. (D and E) The titers of CHIKV-specific antibodies (D) and neutralizing antibodies titers (E) on day 0 and day 28 post-challenge. The dashed lines in panels A, D, E represent the limits of detection. Data represent the mean ± standard deviation (n = 5 in each group). * *p* < 0.05, **p < 0.01, **** *p* < 0.0001.

To determine the *in vivo* protection efficacy of the rAAV-CHIKV-SP against CHIKV infection, the C57BL/6 mice receiving the prime-boost immunizations of rAAV-CHIKV-SP, rAAV-eGFP or unvaccinated PBS controls were challenged with 2.5×10^5^ PFU CHIKV ECSA strain by intradermal footpad injection on day 38 after the primary vaccination, and the viremia and the signs of footpad swelling were analyzed for 3 days and 15 days after challenge, respectively (Fig 2A). As shown in Fig 3A–3C, in the mice from rAAV-eGFP and PBS groups, viremia was induced and lasted 2 days after infection, and obvious footpad swelling with two peaks was observed on day 2 and day 6 post-challenge respectively. This symptom was consistent with that previously reported in CHIKV infected C57BL/6 mice models [24,25]. The first peak of footpad swelling is likely due to the damage caused by virus replication including cell death, cytokine release and tissue edema, and the second prominent peak is associated with the immune-mediated responses including arthritis, tenosynovitis and myositis with marked inflammatory infiltration and muscle damage [24]. In contrast, the rAAV-CHIKV-SP vaccinated mice did not develop viremia, and only showed slight footpad swelling at 1 dpi (Fig 3A–3C), suggesting that the prime-boost immunization of rAAV-CHIKV-SP could provide complete protection against CHIKV infection in C56BL/6 mice. To further investigate the humoral immune responses after challenge, we evaluated the titers of CHIKV-specific antibodies and neutralizing antibodies pre- and post-challenge. As shown in Fig 3D and 3E, by day 28 post-challenge, the CHIKV-specific IgG antibodies and neutralizing antibodies were induced in all the challenged mice, but the titers of the antibodies in the rAAV-CHIKV-SP vaccinated mice were higher than that of the rAAV-eGFP and PBS control mice. Comparing with pre-challenge, the titers of CHIKV-specific IgG antibodies and neutralizing antibodies in rAAV-CHIKV-SP vaccinated mice increased 10-fold (Fig 3D) and 8.4-fold (Fig 3E), respectively, indicating that CHIKV challenge potentiates the antibody responses of the immunized mice.

### A single-dose immunization of rAAV1-CHIKV-SP elicited robust antibody responses in C57BL/6 mice

**Fig 4:**
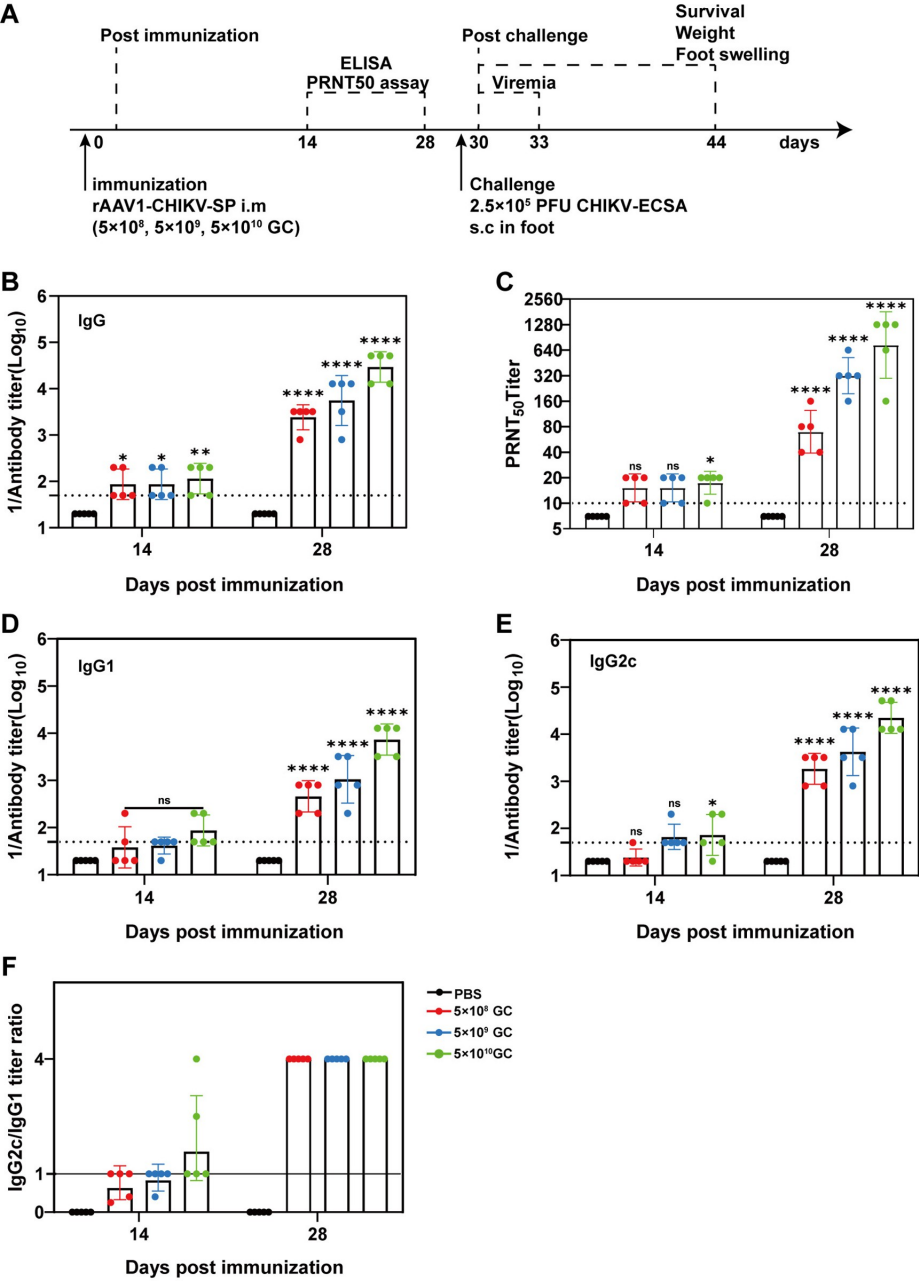
Antibody responses of a single-dose immunization with rAAV1-CHIKV-SP in C57BL/6 mice. (A) Experimental schedule. C57BL/6 mice (4–6 weeks old) were IM injected with different dosages (5 × 10^8^ GC, 5 × 10^9^ GC, or 5 × 10^10^ GC) of rAAV1-CHIKV-SP once. The serum samples on day 14 and 28 post-immunization were subjected to ELISA and PRNT assays. The mice were challenged with 2.5×10^5^ PFU CHIKV ECSA strain virus through s.c. injection in feet on day 30 post-immunization, viremia was determined, and the symptoms post-challenge were recorded at the indicated times post challenge. (B and C) The titers of CHIKV-specific antibodies (B) and neutralizing antibodies (C) from the serum samples determined by ELISA and PRNT, respectively. (D to F) IgG subtype analysis by ELISA. The levels of IgG2c (D) and IgG1 (E) were determined by ELISA, and IgG2c/IgG1 ratios (F) were calculated. Data represent the mean ± standard deviation (n = 5 in each group). * *p* < 0.05, **p < 0.01, **** *p* < 0.0001. n.s., no statistical difference.

With the feature of sustained transgene expression *in vivo*, several rAAV-vectored vaccines have been demonstrated to have capacity to elicit strong and long-term immune responses by single-dose injection [26–28]. To investigate whether a single shot of rAAV1-CHIKV-SP could induce sufficient antibody responses against CHIKV and determine the immunization dose, C57BL/6 mice were given a single injection of three dosages of rAAV1-CHIKV-SP (5×10^8^, 5×10^9^, and 5×10^10^ GC, respectively), and the titers of CHIKV-specific antibodies and the neutralizing antibodies were measured after 14 and 28 days (Fig 4A). Consistent with the prime-boost strategy, the titers of the CHIKV-specific and neutralizing antibodies were at low levels on day 14 and significantly increased by day 28 after immunization (Fig 4B and 4C). The antibody responses induced by rAAV1-CHIKV-SP was dose-dependent, with mice receiving 5×10^10^ GC rAAV1-CHIKV-SP showing the highest levels of CHIKV-specific and neutralizing antibodies. In addition, comparing with the mice immunized with two doses of 5×10^10^ GC rAAV-CHIKV-SP (Fig 2C and 2D), the mice vaccinated once with single-dose of 5×10^10^ GC rAAV1-CHIKV-SP generated similar levels of CHIKV-specific antibodies (1:51,200) and even higher titer of neutralizing antibodies (1:640) (Fig 4B and 4C). The analysis of IgG subtypes showed that, similar with the prime-boost immunization, both IgG1 and IgG2c were elicited, but the IgG2c levels were higher than IgG1 (Fig 4D–4F), further confirming that the rAAV-CHIKV-SP vaccine elicits Th1-polarized immune responses.

### A single-dose of rAAV1-CHIKV-SP protected C57BL/6 mice from CHIKV infection

**Fig 5:**
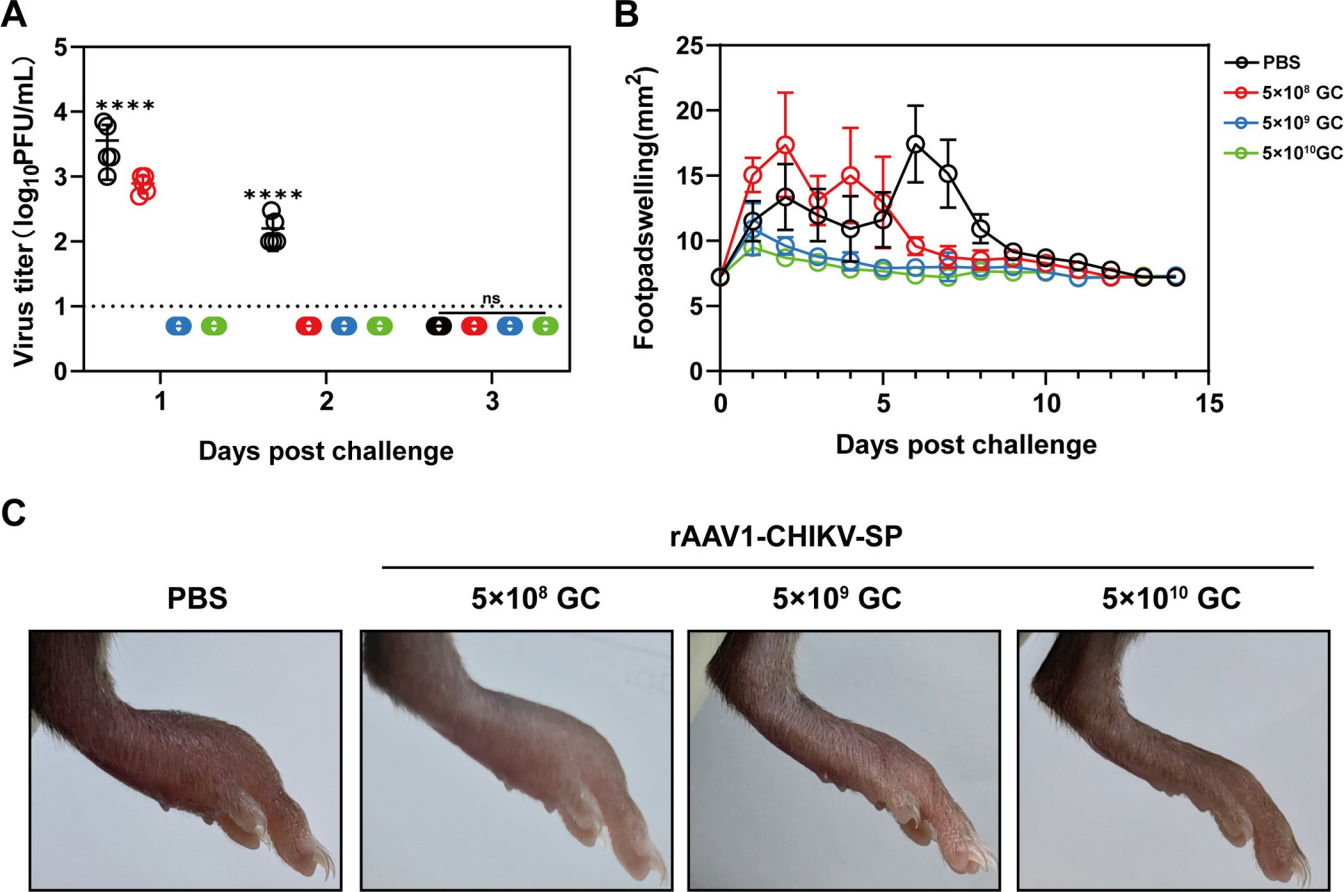
A single-dose immunization with rAAV1-CHIKV-SP protects C57BL/6 mice from CHIKV infection. (A) The viremia of mice receiving single-dose injection of 5 × 10^8^ GC, 5 × 10^9^ GC, or 5 × 10^10^ GC of rAAV1-CHIKV-SP respectively in 3 days post-challenge. (B) The footpad swelling measurements for 15 days after challenge. (C) Representative images of the injected feet of the mice from each group on day 5 post-challenge. The dashed lines in panels A represent the limit of detection. Data represent the mean ± standard deviation of 5 mice at each time point in each group. The asterisks denote statistical differences between the indicated groups. **** *p* < 0.0001; n.s., no statistical difference.

After verifying the strong immunogenicity of a single-dose of rAAV1-CHIKV-SP, we next evaluated the protection efficacy of a single injection of different dosages of rAAV1-CHIKV-SP. The C57BL/6 mice receiving a single dose immunization of 5×10^8^, 5×10^9^, and 5×10^10^ GC rAAV1-CHIKV-SP were challenged with 2.5×10^5^ PFU CHIKV-ECSA viruses on day 30 post-vaccination, and the viremia and foot swelling conditions were monitored after challenge (Fig 4A). As shown in Fig 5, mice receiving the dosages of 5×10^9^ and 5×10^10^ GC rAAV1-CHIKV-SP completely controlled the virus challenge with no detectable viremia and significant foot swelling after the virus attack. Meanwhile, mice immunized with 5×10^8^ GC rAAV1-CHIKV-SP were partially protected, showing reduced levels of viremia and earlier recovery of the foot swelling comparing with the PBS control group (Fig 5). These results suggest that a single-dose of rAAV1-CHIKV-SP could provide protection efficacy against CHIKV infection with a dosage-dependent pattern in C57BL/6 mice.

### A single-dose of rAAV1-CHIKV-SP induced long-term antibody responses in C57BL/6 mice

**Fig 6:**
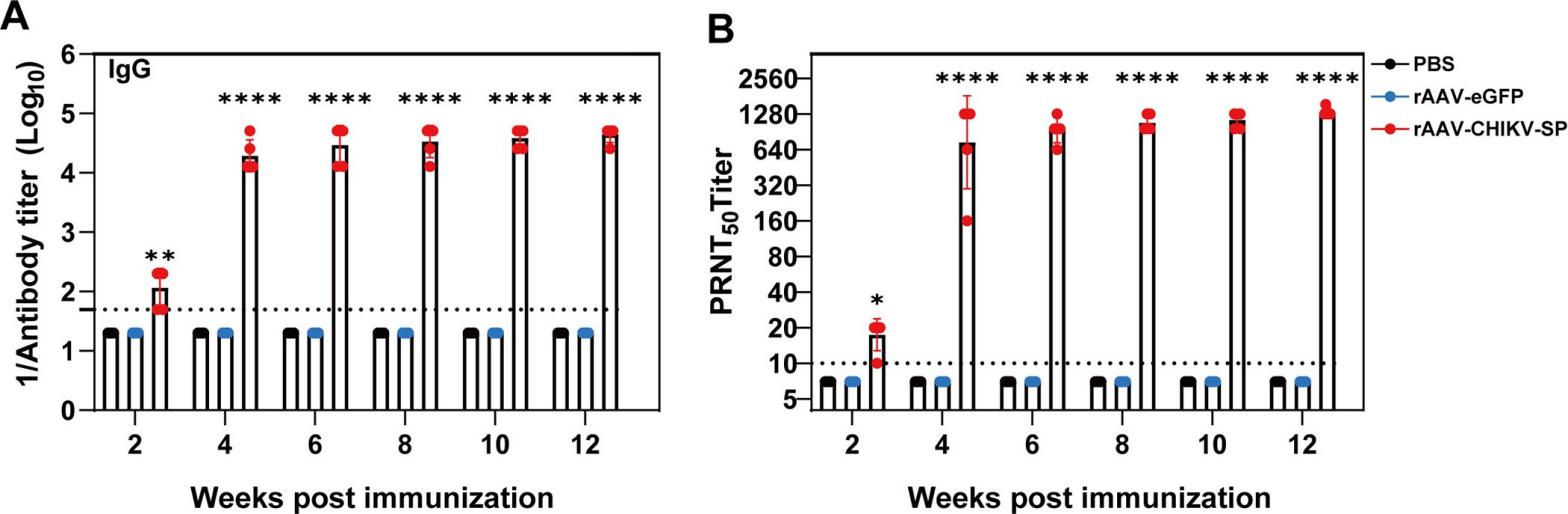
Long-term antibody responses induced by a single-dose of rAAV1-CHIKV-SP. Groups of 4–6 weeks old C57BL/6 mice were given a single-dose IM injection of 5 × 10^10^ GC rAAV1-CHIKV-SP or rAAV1-eGFP or PBS. The serum samples of mice from each group were collected at 2, 4, 6, 8, 10 and 12 weeks after immunization, and subjected to ELISA and PRNT assays to quantify the levels of CHIKV-specific antibodies (A) and neutralizing antibodies (B), respectively. The dashed lines represent the limit of detection. Data represent the mean ± standard deviation of 5 mice at each time point in each group. The asterisks denote statistical differences between the indicated groups. * *p* < 0.05, **p < 0.01, **** *p* < 0.0001.

To evaluate the longevity of rAAV1-CHIKV-SP induced antibody responses, a group of C57BL/6 mice inoculated with a single-dose of 5×10^10^ GC rAAV1-CHIKV-SP were not challenged and maintained for 12 weeks, and the titers of CHIKV-specific and neutralizing antibodies were continuingly analyzed at two-week intervals post-immunization. As shown in Fig 6A and 6B, the CHIKV-specific IgG and the neutralizing antibodies increased to their peak titers of 1:51,200 at week 6 and 1: 1,280 at week 8, respectively, and both of them remained at a high level thereafter. Furthermore, all the rAAV1-CHIKV-SP immunized mice showed no adverse symptoms throughout the experimental period, further confirming the safety profile of the vaccine. These results suggest that a single-dose immunization of rAAV1-CHIKV-SP vaccine can induce robust and long-term antibody responses against CHIKV infection.

## Discussion

Due to the influence of climate change, increased urbanization and frequent global travel, the mosquito-transmitted CHIKV virus has greatly expanded its distribution range and become a major global health threat, necessitating the development of vaccines. The recombinant AAV vectors have shown excellent safety and efficacy in preclinical and clinical settings for various gene therapies, attracting increasing number of studies exploring their use as a vaccine platform. Several AAV vector-based vaccines against infectious diseases have shown strong immunogenicity and effective protection against viral infection in pre-clinical studies. However, whether AAV vectors can be used for the development of vaccines against alphaviruses remains unknown. In this study, we had evaluated the potential of the AAV vector to develop a safe and effective CHIKV vaccine.

The neutralizing antibodies play essential role in the control of CHIKV infection [29,30]. Previous studies have demonstrated that the E2 protein is the dominant target for the neutralizing antibodies in infected patients and individuals vaccinated with a candidate CHIKV vaccine [31,32]. However, the single expression of E2 protein or combination expression with E3 or E1 by the modified vaccinia virus Ankara (MVA) and adenovirus vectors only induced low levels of neutralizing antibodies, whereas the expression of the complete structural proteins (Capsid-E3-E2-6K-E1) or the entire envelop proteins (E3-E2-6K-E1) elicited significantly higher levels of neutralizing antibodies [33–35]. One possible explanation for the higher immunogenicity of the Capsid-E3-E2-6K-E1 is its ability to produce the secreted virus-like particles (VLPs) that mimic the external structure of wild-type virions and further enhance the humoral immune response. Accordingly, the DNA plasmid or measles virus vectors encoding the full-length structural proteins led to the release of VLPs from the transfected or infected cells, and were capable to induce potent neutralizing antibody responses [36–38]. Therefore, we chose the Capsid-E3-E2-6K-E1 polyprotein as the antigen. However, we had detected no presence of VLPs in the supernatants harvested either from rAAV-CHIKV-SP producing cells or infected cells with no detectable expression of E2 by Western blot analysis (S1 Fig). Possible explanations for the absence of VLPs may be that the polyprotein may not have been adequately processed as we didn’t examine the expression and processing of other structural proteins due to lack of their antibodies, and the low transgenic expression efficiency of rAAV vectors *in vitro* [39] may also affect the VLPs production. However, we still observed strong immunogenicity for rAAV-CHIKV-SP that a single dose immunization induced high levels of both CHIKV-specific and neutralizing antibodies. Similarly, a recombinant MVA encoding the CHIKV Capsid-E3-E2-6K-E1 also failed to produce detectable VLPs, but was highly immunogenic in C57BL/6 mice [33], indicating the superior immunogenicity of the full-length structural proteins regardless of VLPs formation.

In our previous studies, we had developed an attenuated ΔC-CHIKV with the entire capsid gene deletion in the CHIKV genome [20] and an optimized chimeric VEEV-ΔC-CHIKV attenuated vaccine candidate subsequently [40]. By single-dose immunization, the rAAV1-CHIKV-SP induced lower antibody levels at 14 days, but higher antibody titers at 28 days after vaccination comparing with these two attenuated vaccines. The lag of immune responses of AAV-vectored vaccines had also been reported in the studies of rAAV-based Nipah virus and rabies virus vaccines [15, 28], possibly due to the delayed transgene expression of rAAV vectors [41]. With the continuous expression of the AAV-vectored antigen, potent immune response will be elicited and maintained at a high level. Therefore, the rAAV-based vaccines may take longer time to build strong protection, but a single-dose immunization is sufficient to elicit sufficient and persistent immune responses against the antigens, just as has been reported in AAV-vectored HCV, influenza, SARS-CoV-2 and rabies virus vaccines [14, 27, 28, 42]. In this study, we also found that a single-dose injection of 5×10^10^ GC rAAV1-CHIKV-SP elicited comparable antibody responses with the two-dose vaccination of 5×10^10^ GC rAAV1-CHIKV-SP and 5×10^10^ GC rAAV9-CHIKV-SP, and sustained long-lasting neutralization titers. Similarly, in a previous study of AAV8 vectored Nipah virus vaccine, a boost injection with an alternative AAV serotype didn’t significantly enhance the antibody responses [15]. Several studies have demonstrated that a heterologous vaccine such as adenovirus or DNA based vaccine boost could increase the AAV-based vaccine induced immune responses [43, 44], suggesting that a heterologous CHIKV vaccine platform would possibly be beneficial to enhance the protection effect of rAAV1-CHIKV-SP.

We found that rAAV-CHIKV-SP immunization in C57BL/6 mice generated a Th1-skewed immune response with the IgG2c/IgG1 ratio > 1. Previous studies have suggested that the ability to induce Th1-biased immune responses are beneficial for the vaccines against virus pathogens, since the Th1 response is crucial for eliminating intracellular virus infection, and can avoid the excessive activation of Th2 response which may increase the risk of vaccine-induced enhancement of the diseases following infection [45, 46]. However, multiple factors may influence the immune responses of different vaccine candidates such as the virus strains, the administration routes, the adjuvants, and the animal models in the experimental system. For example, the VLP vaccines have been reported to induce either a balanced Th1/Th2 response or Th1 dominant immune response by two different studies [37, 47]. Notably, the C57BL/6 mice we used are characterized to exhibit Th1-type immune response [48]. Therefore, although the results indicated a Th1-biased immune response, further studies are needed to evaluate the quality of the immune responses induced by rAAV-CHIKV-SP.

In this study, the antigen of the rAAV-CHIKV-SP vaccine was derived from a CHIKV Asian strain [17], and the sera from the immunized mice exhibited potent neutralizing activities against the wild type CHIKV Asian strain in the PRNT assay (Figs 2 and 4). A heterologous CHIKV ECSA virus challenge model was used to evaluate the protection effect of the vaccine. In this model, C57BL/6 mice are inoculated with the CHIKV ECSA viruses through subcutaneous injection in the footpad, and the infected mice develop high levels of viremia and significant footpad swelling [20]. Previous studies demonstrated that the swelling feet of the CHIKV infected C57BL/6 mice showed clear signs of arthritis, tenosynovitis and myositis that are consistent with acute CHIKV infection in humans, and the level of footpad swelling associates with the level of inflammatory infiltrations and proinflammatory mediators [24, 25]. All the immunized mice were cross-protected from the CHIKV ECSA virus challenge. Previous studies demonstrated that most neutralizing antibodies bind to the A and B domains of CHIKV E2 [6]. The sequences of the A and B domains are conserved between the CHIKV Asian and ECSA strains. Only one variation (E118) in A domain, and three variations (E2-195, E2-205 and E2-207) in B domain were observed, and these variations are not within the residues crucial for the binding of broad neutralizing monoclonal antibodies [6], which may explain the cross-protective effect of rAAV1-CHIKV-SP induced immune response against the two different strains.

We found that the mice received a single-dose IM injection of rAAV-CHIKV-SP higher than 5×10^9^ GC (approximately 2.5×10^11^ GC/kg) were completely protected. A previous clinical trial has suggested that AAV1 doses up to 1.2×10^14^ GC (about 2 ×10^12^ GC/kg) administered by IM injection are safe in healthy adults [49], implying the safety of rAAV-CHIKV-SP vaccine for use in humans. However, although AAV1 vector has been approved for application in humans, there are still several challenges restricting its clinical application. Both humoral and CD8^+^ T cell responses against the AAV1 capsid were induced in the volunteers in the clinical trial [49], and the prevalence of anti-AAV1 antibodies is close to 74.9% of the adult population [50], these pre-existing immune responses may compromise the gene transfer efficacy of AAV vectors, thereby posing limitations of the applicable population and re-administration of rAAV. Another major hindrance for rAAV application is the relatively limited capability for large-scale and economically viable manufacturing. Numerous efforts have been made to explore approaches to overcome the pre-existing immunity and develop advanced production platforms enabling scalable production, and have shown many promising results [51]. With the continuous effort to develop and optimize the rAAV systems, these challenges are expected to be addressed, thereby improving the practicality of AAV vectors and expanding their clinical applications.

In summary, our data revealed the potential of the rAAV based vaccine to combat CHIKV. A single and low dose of the vaccine induced substantial and long-lasting antibody responses, and provide complete protection from CHIKV challenge in C57BL/6 mice model. However, there are several limitations in this study that need to be solved in our future researches. First, further evaluation of the vaccine in male C57BL/6 mice is necessary, as previous studies have demonstrated that the biological gender affects the immune responses of mice to viruses or vaccines [52,53]. The mechanism behind the protection of the vaccine had not been fully identified. The neutralizing activities of the antibodies induced by the vaccine were examined using the *in vitro* PRNT assay, but the real protective role of the antibodies *in vivo* needs to be further confirmed by passive transfer experiment of the immune serum to naïve mice. In addition, this study used an ELISA assay with inactivated CHIKV with surface display of E2/E1 heterodimers as antigen, which could not distinguish between antibodies targeting E2 and E1 respectively. To investigate the immunogenicity of each structural protein, ELISA assay based on soluble recombinant proteins should be established in the future study. In contrast to the known protective humoral immunity, the contribution of CD8^+^ T cells in the control of CHIKV remains unclear. A previous study showed that the T cells elicited by a live attenuated CHIKV/IRES vaccine did not confer protection against CHIKV infection [54], while using the peptides only elicited robust T cell responses but bot neutralizing antibodies, another study demonstrated a protective role of CD8^+^ T cells in CHIKV infection [55]. Previous studies had demonstrated that the AAV vectored SARS-CoV-2 vaccines could elicit strong antigen-specific T cell response [26,56]. It is significant to analyze whether rAAV1-CHIKV-SP can trigger CHIKV-specific T cell immune response and evaluate its role in antiviral response. Overall, this work is a proof-of-concept study for rAAV1-CHIKV-SP as a potential vaccine against CHIKV, extensive studies including the optimization and scale-up of production of the vaccine, as well as pre-clinical studies on the safety and efficacy of the vaccine in other animal models are required to facilitate clinical development of the vaccine.

## Supporting information

S1 Fig The detection of the CHIKV-E2 protein in the supernatants of rAAV-CHIKV-SP transfected cells and infected cells. (DOCX)

S1 Raw Data The raw data of Fig 1B–1F, 2B–2G, 3A–3E, 4B–4F, 5A–5C, 6A–6B, and S1 Fig of this study. (ZIP)
