# Supplementary material for: A single dose recombinant AAV based CHIKV vaccine elicits robust and durable protective antibody responses in mice
Source: PLoS Negl Trop Dis. 2024 Nov 4;18(11):e0012604. doi: 10.1371/journal.pntd.0012604 (PMC11563480; doi:10.1371/journal.pntd.0012604)
Supplement: S1 Fig — (DOCX) [file pntd.0012604.s001.docx]

**A single dose recombinant AAV based CHIKV vaccine elicits robust and durable protective antibody responses in mice**

Qin-Xuan Zhu ^1†^, Ya-Nan Zhang ^2†^, Hong-Qing Zhang ^2,3^, Chao Leng ^2^, Cheng-Lin Deng ^2^, Xin Wang ^1^, Jia-Jia Li ^1^, Xiang-Li Ye ^1^, Bo Zhang ^2*^, Xiao-Dan Li ^1*^

^1^ Hunan Normal University, School of Medicine, Changsha, 410081, China

^2^ Key Laboratory of Special Pathogens and Biosafety, Wuhan Institute of Virology, Center for Biosafety Mega-Science, Chinese Academy of Sciences, Wuhan, 430207, China;

^3^ University of Chinese Academy of Sciences, Beijing 100049, China

^†^ These authors have equal contribution to this work.

Corresponding author: Xiao-Dan Li, E-mail: [lxd@live.cn](mailto:lxd@live.cn); Bo Zhang, E-mail: [zhangbo@wh.iov.cn](mailto:zhangbo@wh.iov.cn)

**Supplementary Materials**

**S1 Fig**


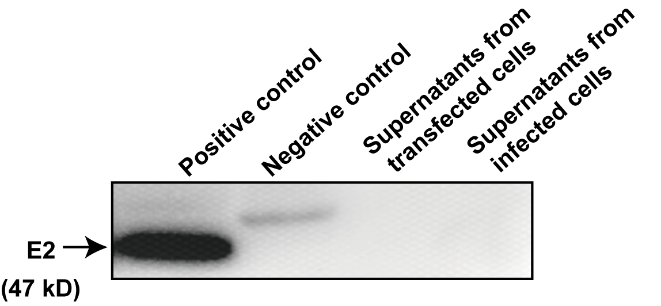


**S1 Fig. The detection of the CHIKV-E2 protein in the supernatants of rAAV-CHIKV-SP transfected cells and infected cells.** The supernatants harvested from pAAV-CHIKV-SP, AAV1 packaging-plasmid and pHelper co-transfected cells at 72 hpt, and the supernatants harvested from the rAAV-CHIKV-SP infected cells (MOI=1×10^5^) at 72 hpi, were filtered through a 0.22 μm microporous membrane and subjected to Western blot assay using the CHIKV E2 polyclonal antiserum as primary antibody. The cell lysis of the CHIKV-WT infected cells and HEK-293T mock were used as positive and negative controls, respectively.
