# Supplementary figures and images for: A single dose recombinant AAV based CHIKV vaccine elicits robust and durable protective antibody responses in mice
Source: PLoS Negl Trop Dis. 2024 Nov 4;18(11):e0012604. doi: 10.1371/journal.pntd.0012604 (PMC11563480; doi:10.1371/journal.pntd.0012604)

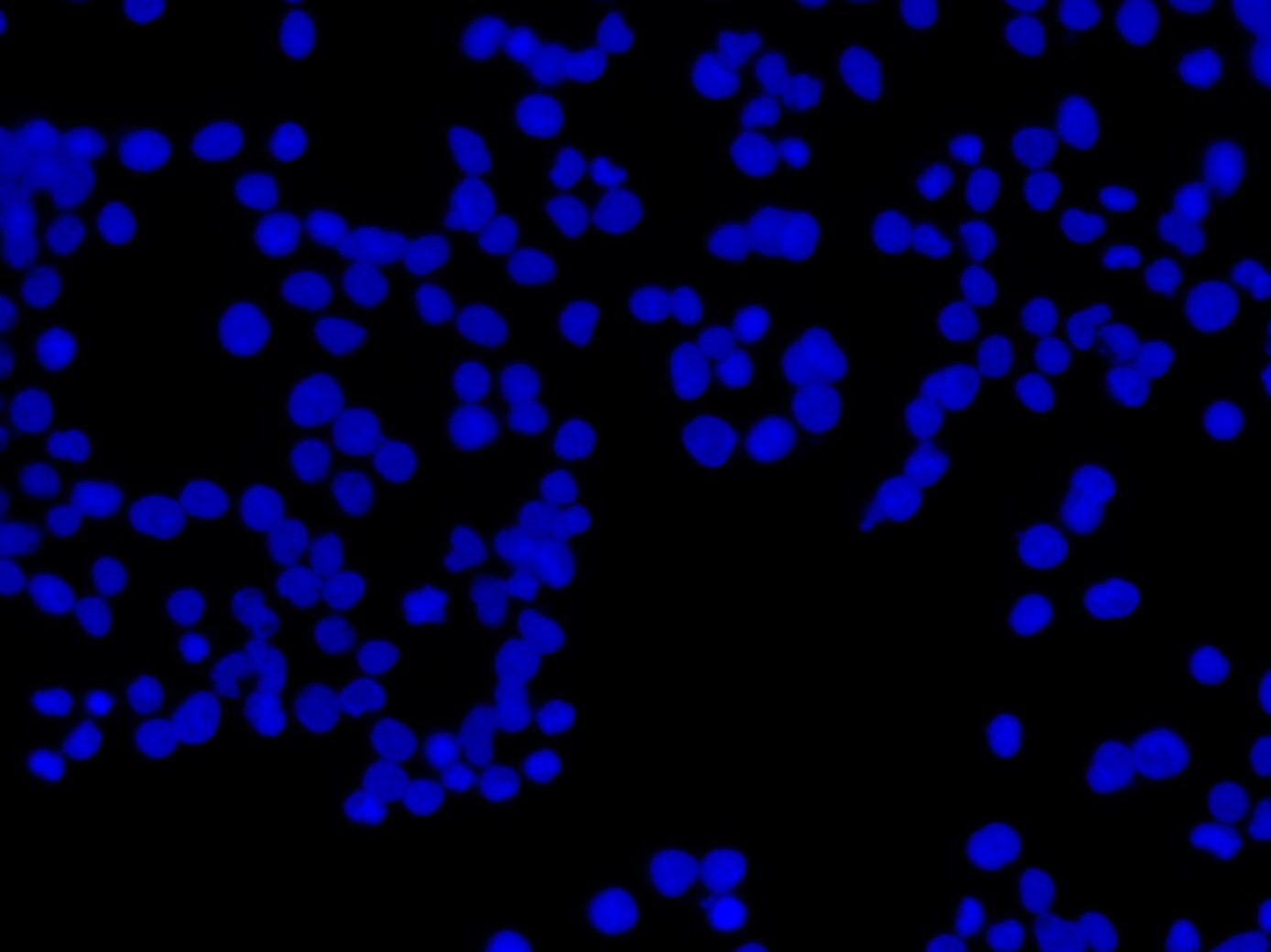

Supplement: S1 Raw Data — (ZIP) [file pntd.0012604.s002.zip › S1_RawData/Raw data/Fig 1/Fig 1B/mock/DAPI.JPG]

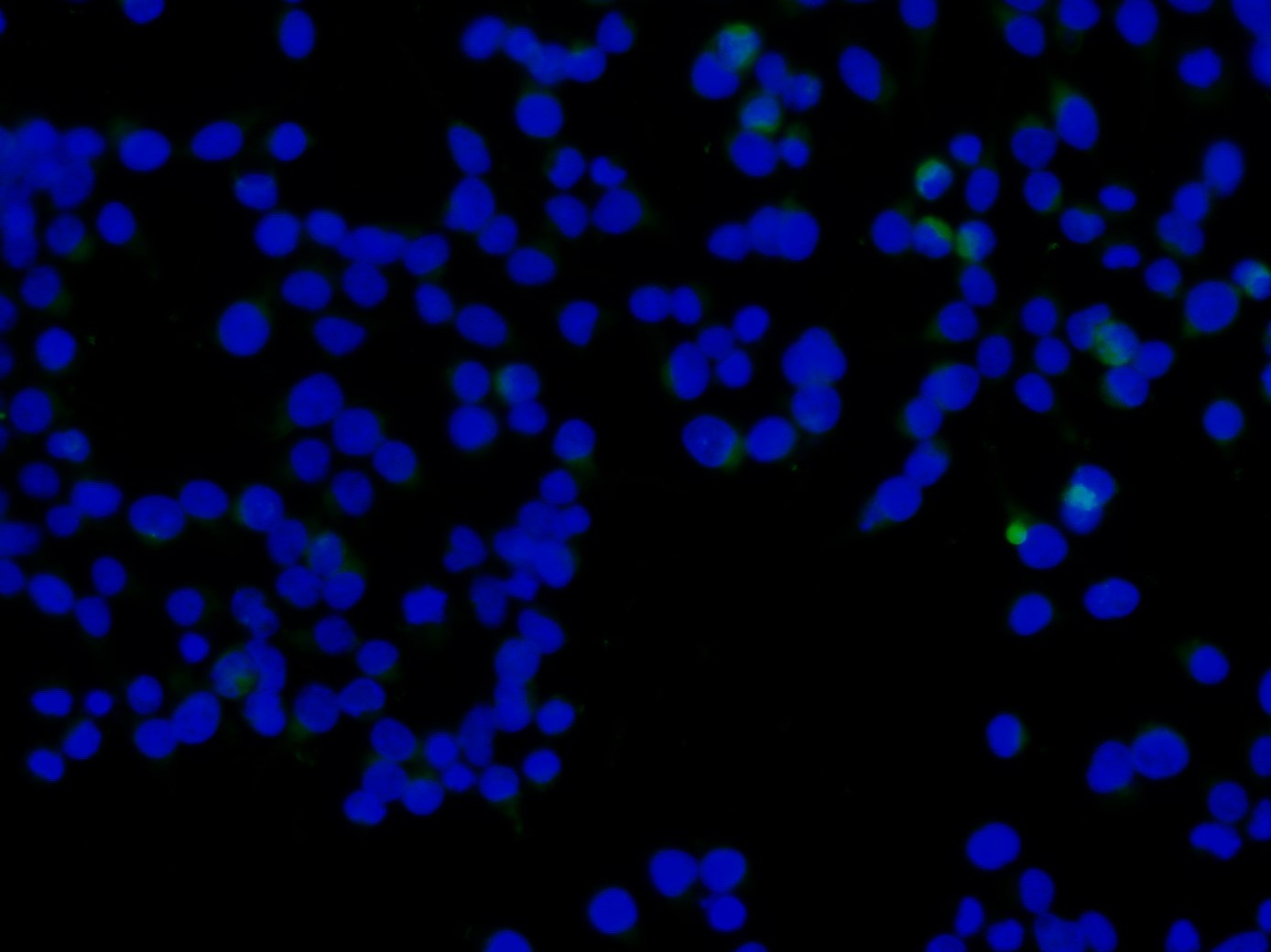

Supplement: S1 Raw Data — (ZIP) [file pntd.0012604.s002.zip › S1_RawData/Raw data/Fig 1/Fig 1B/mock/Merge.JPG]

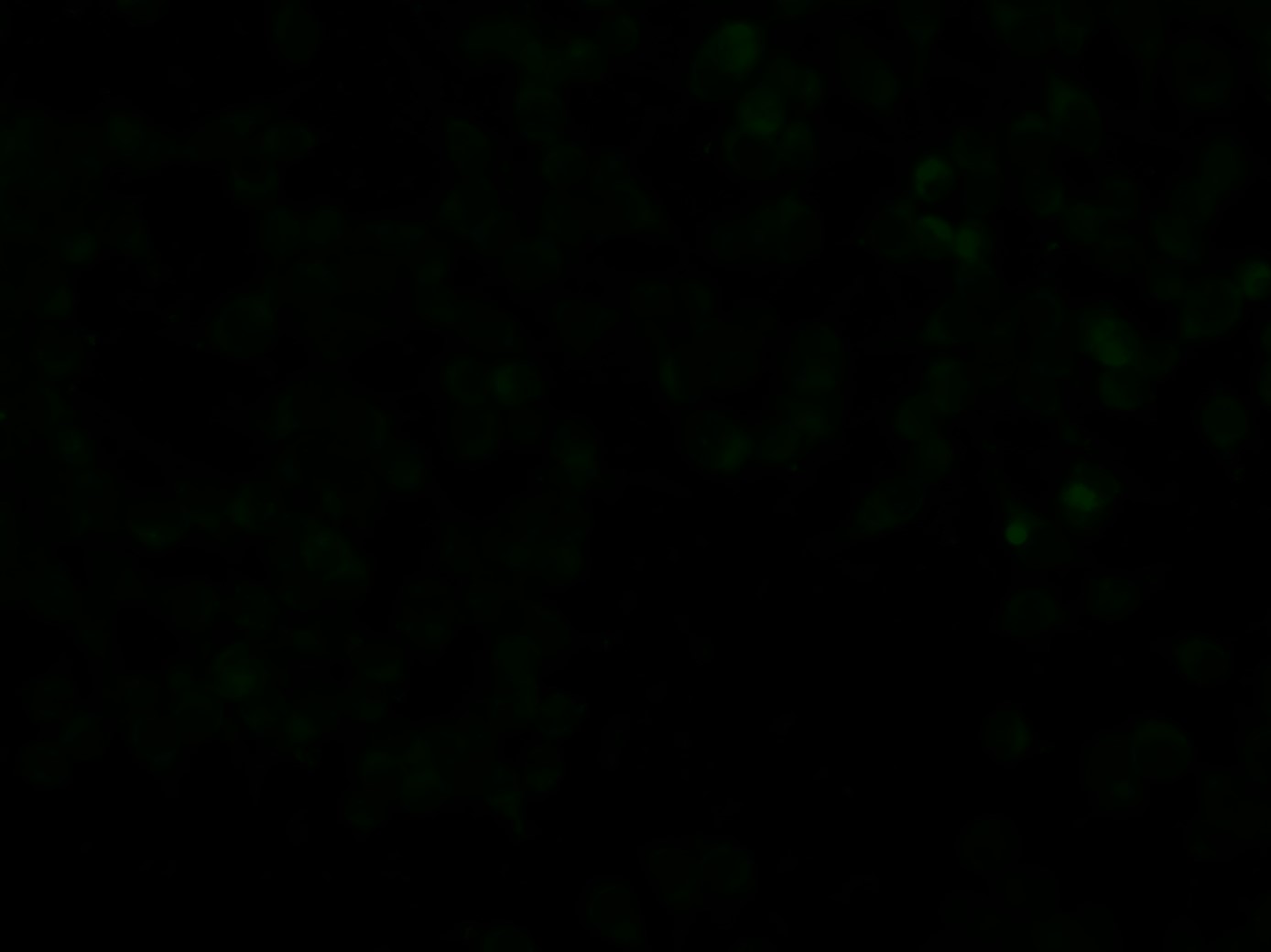

Supplement: S1 Raw Data — (ZIP) [file pntd.0012604.s002.zip › S1_RawData/Raw data/Fig 1/Fig 1B/mock/mock-E2.JPG]

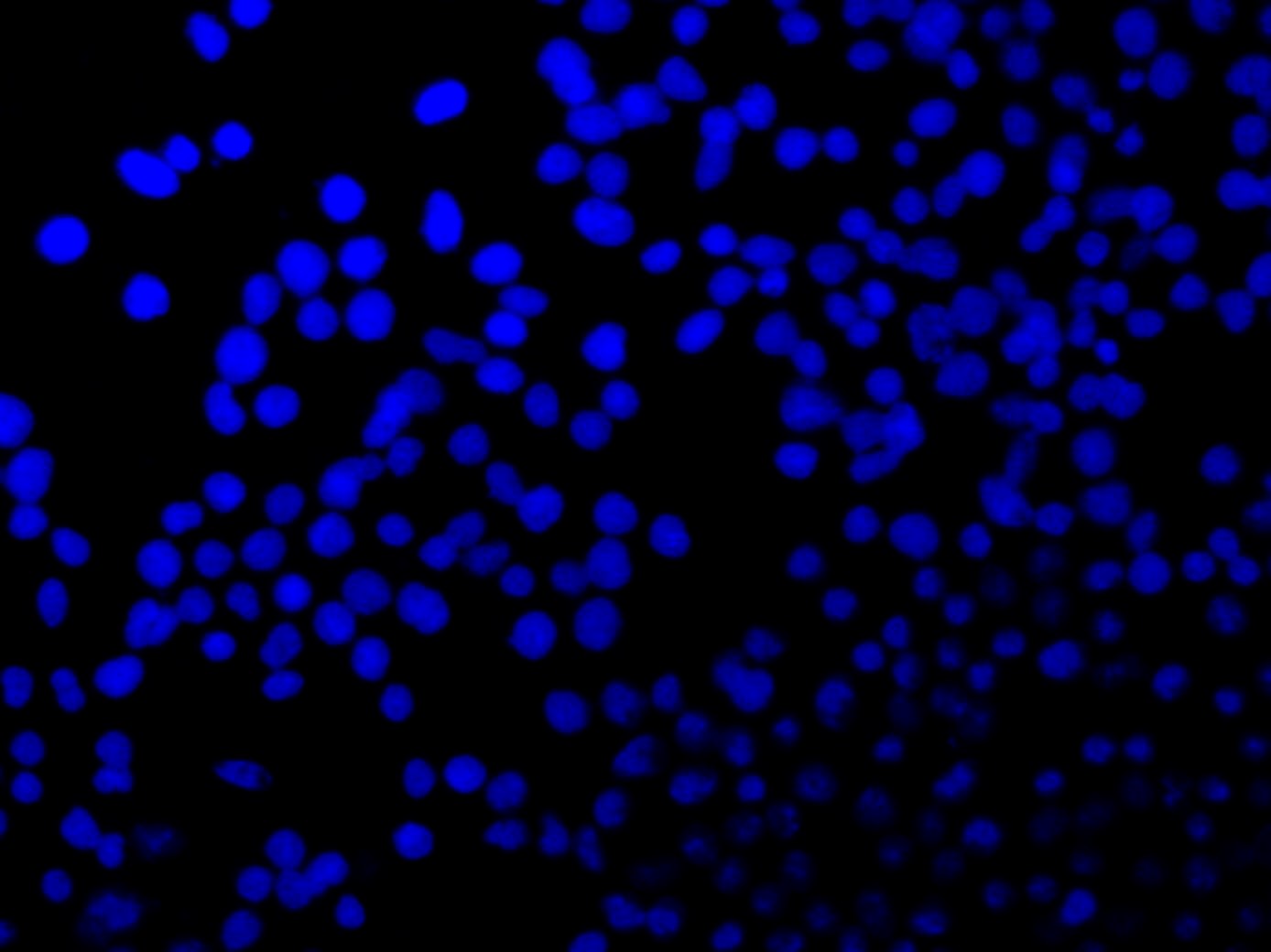

Supplement: S1 Raw Data — (ZIP) [file pntd.0012604.s002.zip › S1_RawData/Raw data/Fig 1/Fig 1B/pAAV-CHIKV-SP/DAPI.JPG]

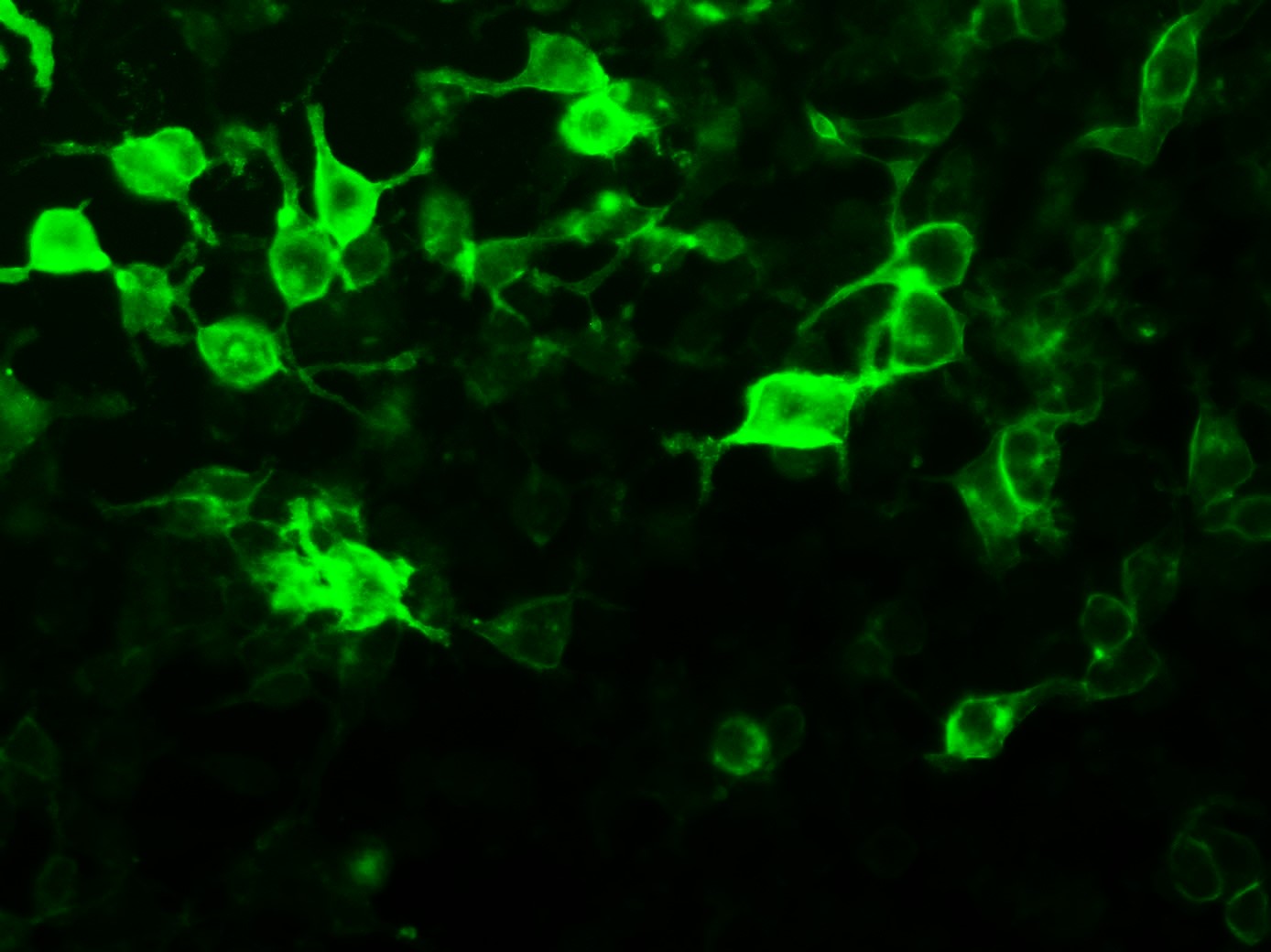

Supplement: S1 Raw Data — (ZIP) [file pntd.0012604.s002.zip › S1_RawData/Raw data/Fig 1/Fig 1B/pAAV-CHIKV-SP/E2.JPG]

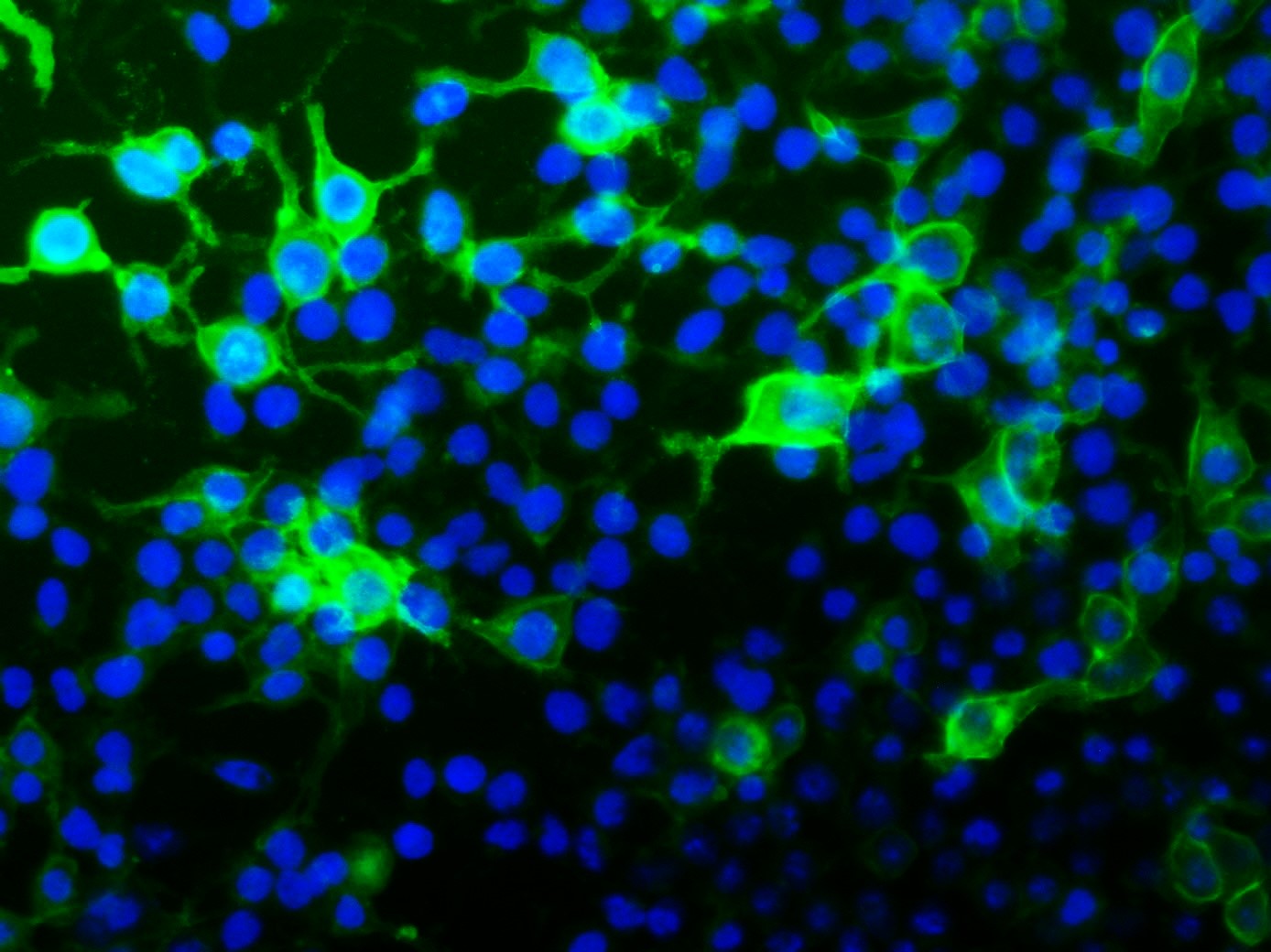

Supplement: S1 Raw Data — (ZIP) [file pntd.0012604.s002.zip › S1_RawData/Raw data/Fig 1/Fig 1B/pAAV-CHIKV-SP/merge.JPG]

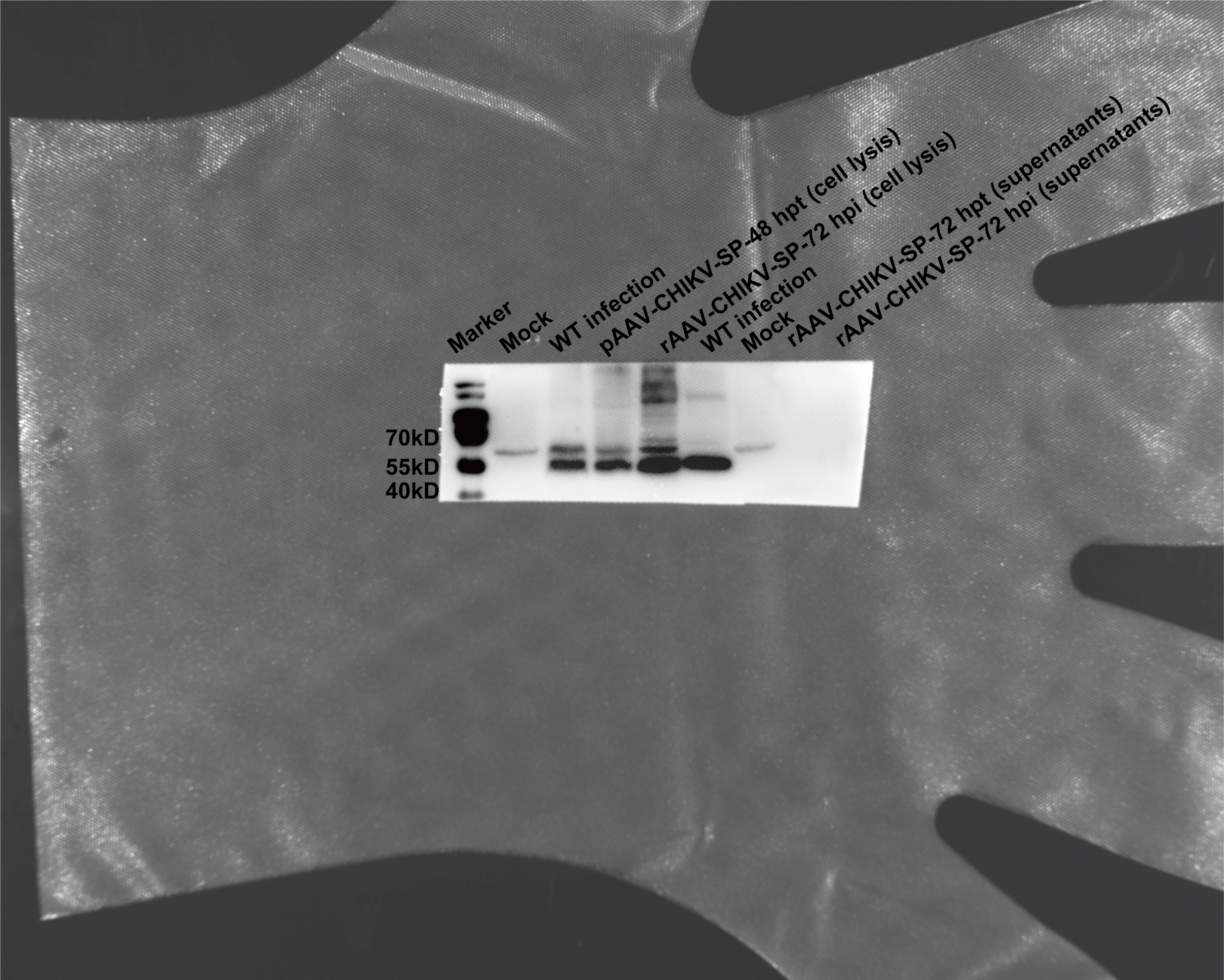

Supplement: S1 Raw Data — (ZIP) [file pntd.0012604.s002.zip › S1_RawData/Raw data/Fig 1/Fig 1C/rAAV-CHIKV-SP- WB.tif]

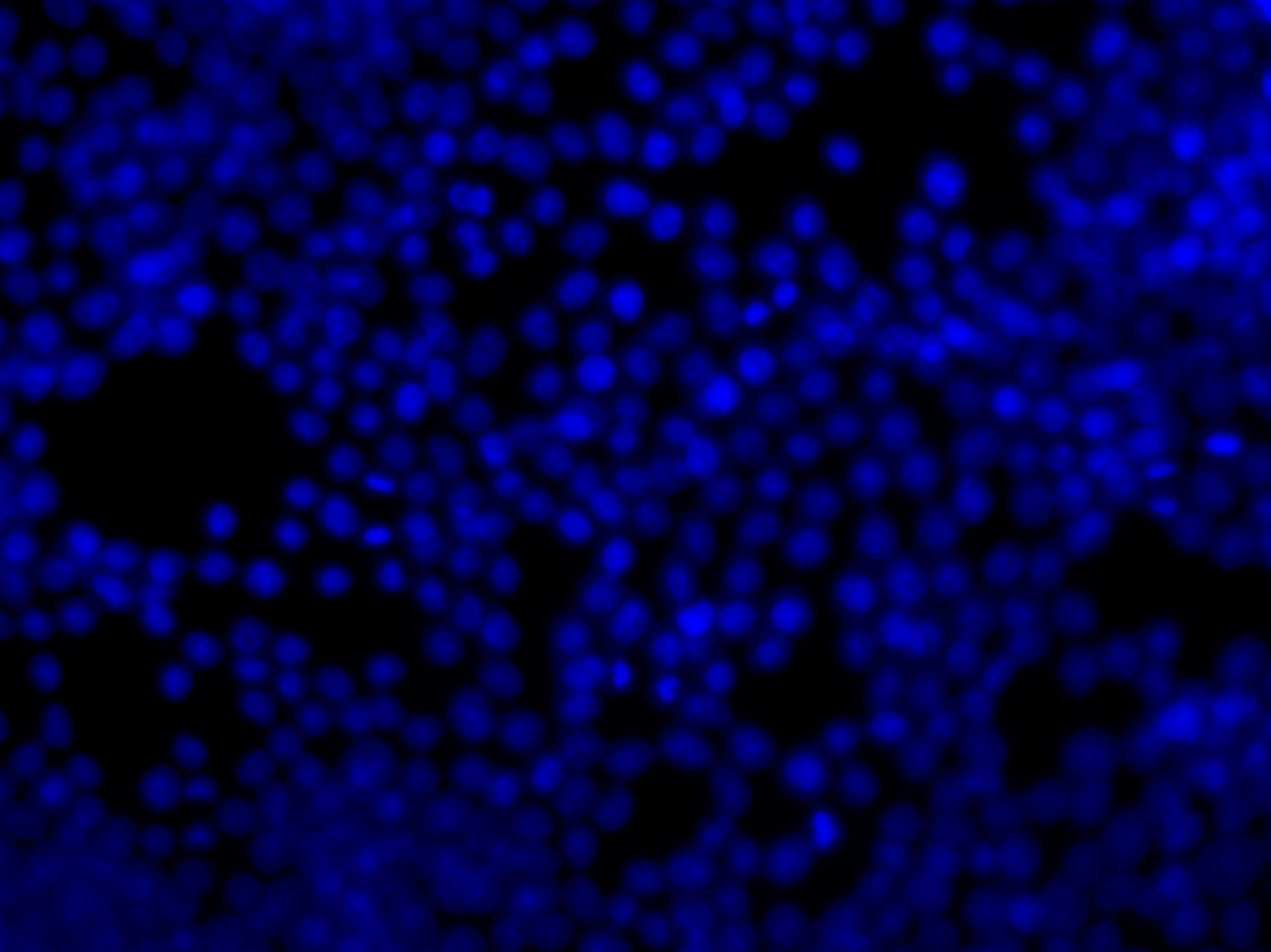

Supplement: S1 Raw Data — (ZIP) [file pntd.0012604.s002.zip › S1_RawData/Raw data/Fig 1/Fig 1E/MOCK/DAPI.JPG]

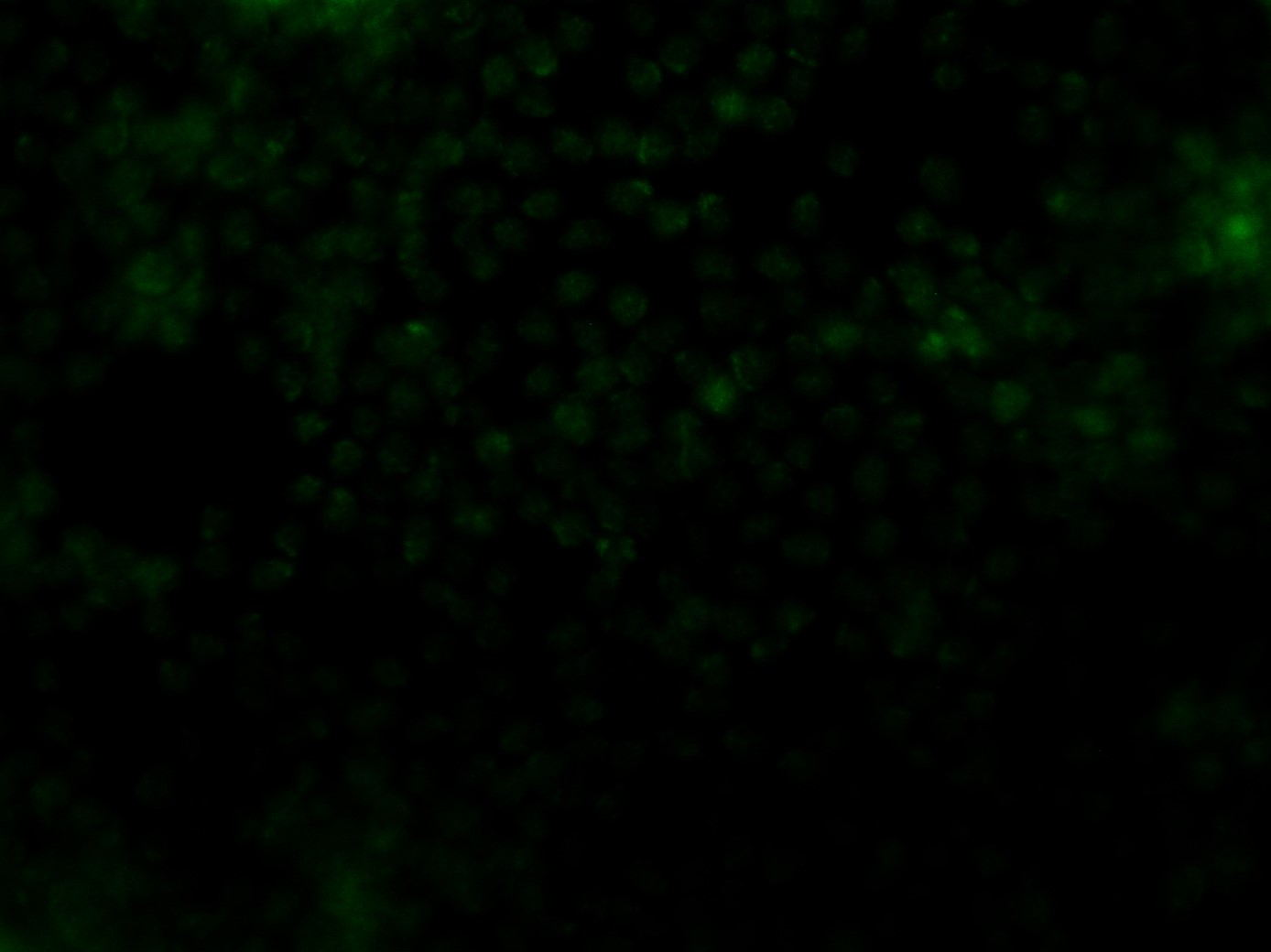

Supplement: S1 Raw Data — (ZIP) [file pntd.0012604.s002.zip › S1_RawData/Raw data/Fig 1/Fig 1E/MOCK/E2.JPG]

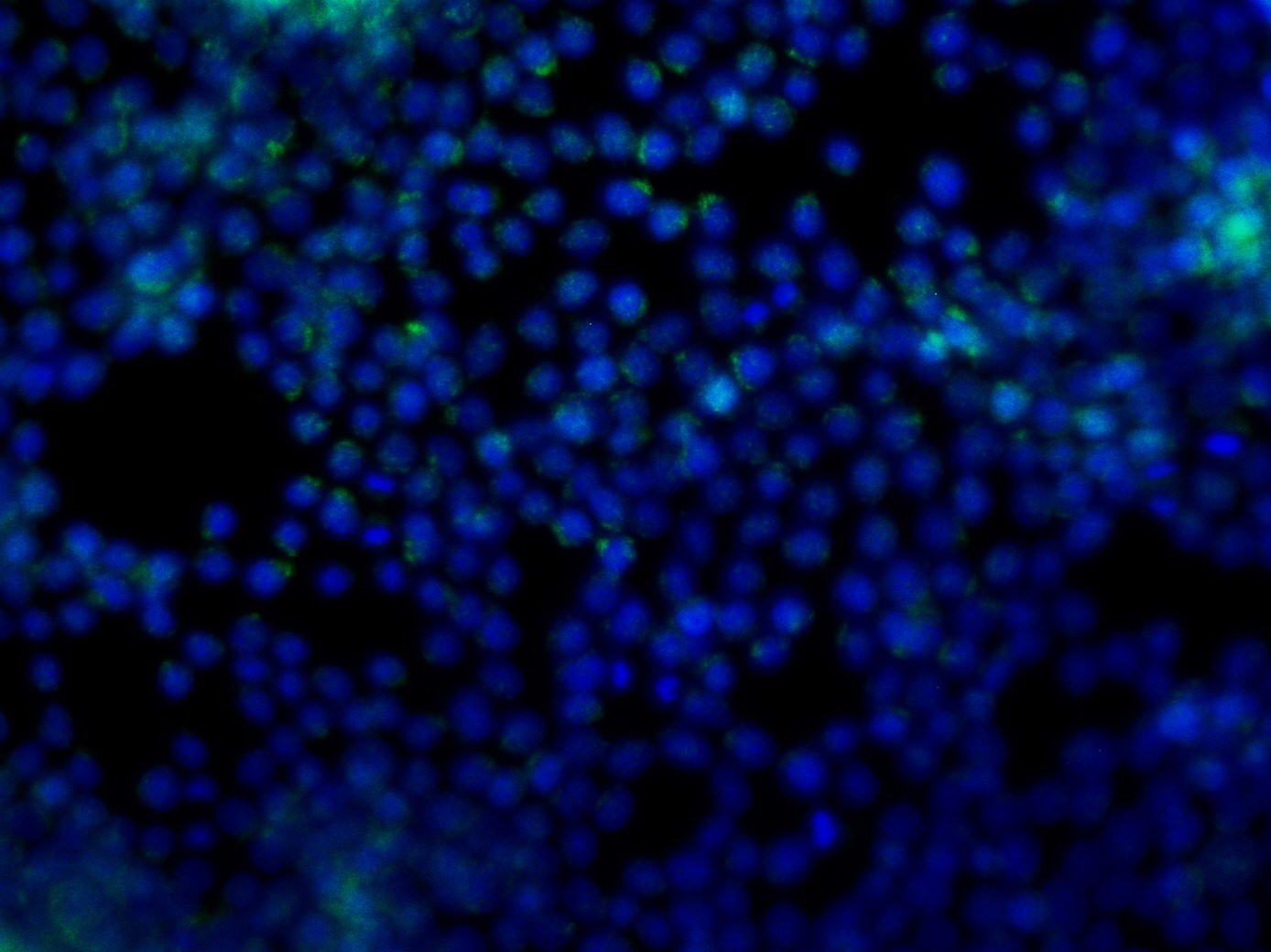

Supplement: S1 Raw Data — (ZIP) [file pntd.0012604.s002.zip › S1_RawData/Raw data/Fig 1/Fig 1E/MOCK/merge.JPG]

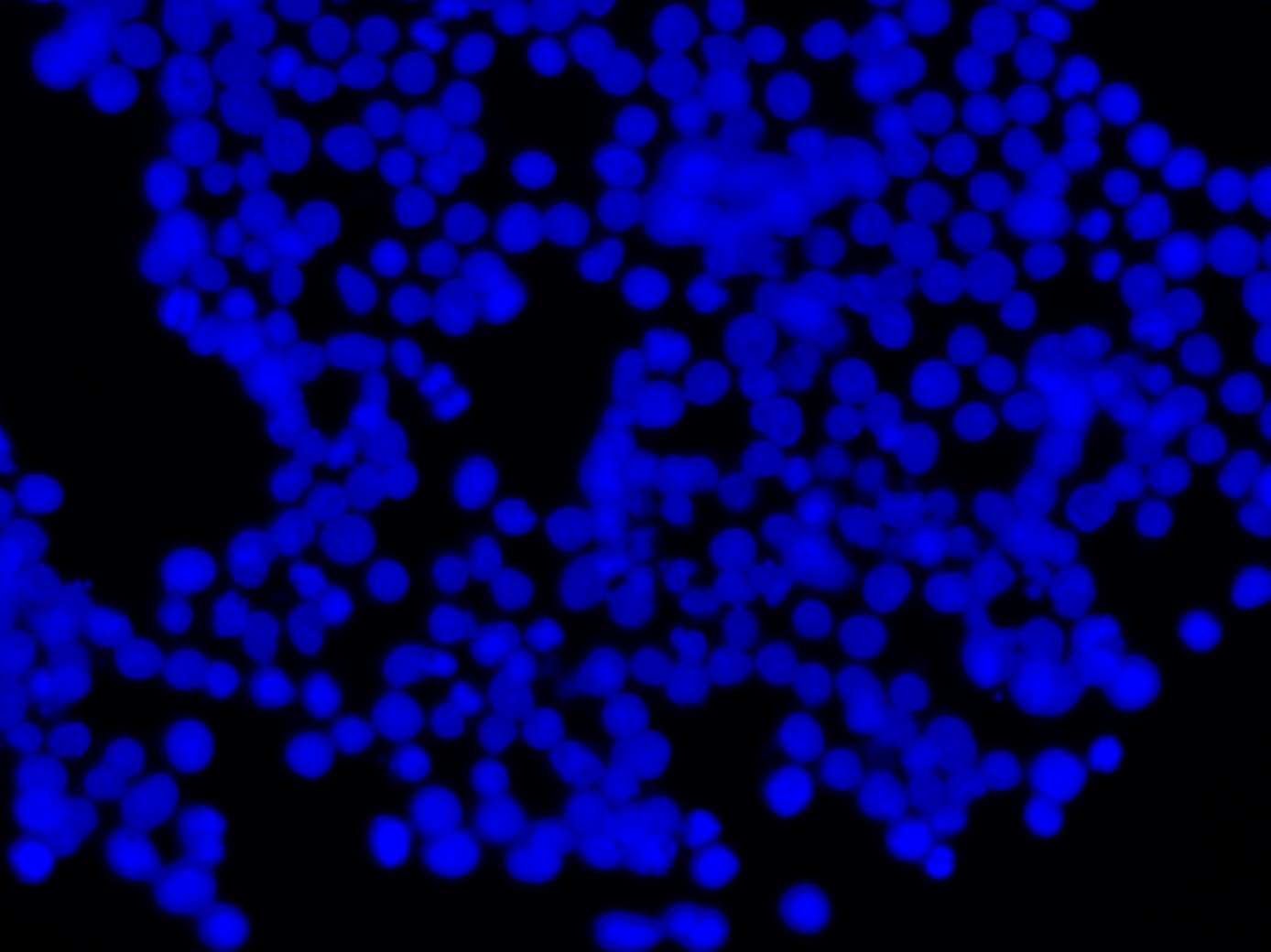

Supplement: S1 Raw Data — (ZIP) [file pntd.0012604.s002.zip › S1_RawData/Raw data/Fig 1/Fig 1E/rAAV-CHIKV-SP/DAPI.JPG]

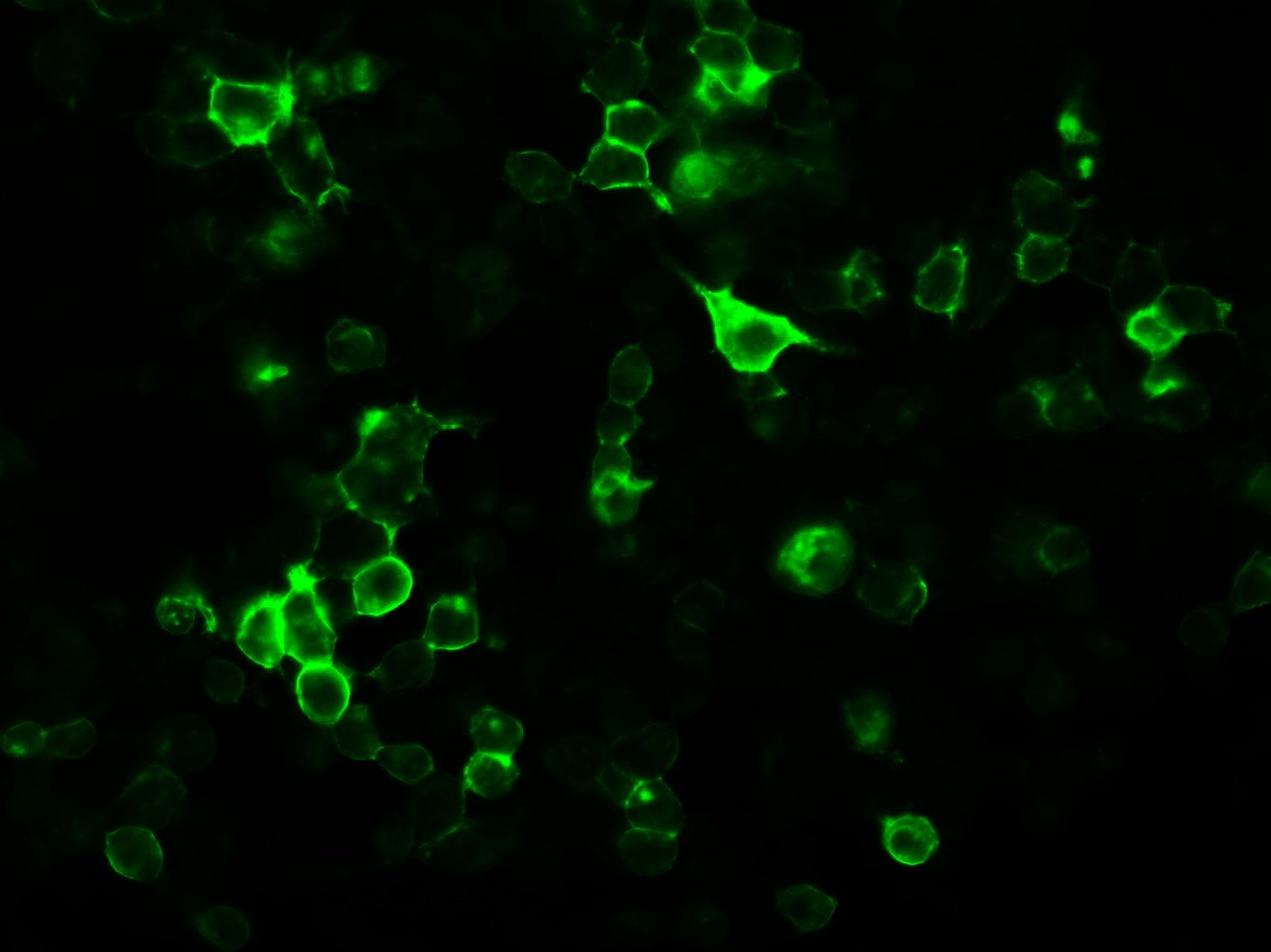

Supplement: S1 Raw Data — (ZIP) [file pntd.0012604.s002.zip › S1_RawData/Raw data/Fig 1/Fig 1E/rAAV-CHIKV-SP/E2.JPG]

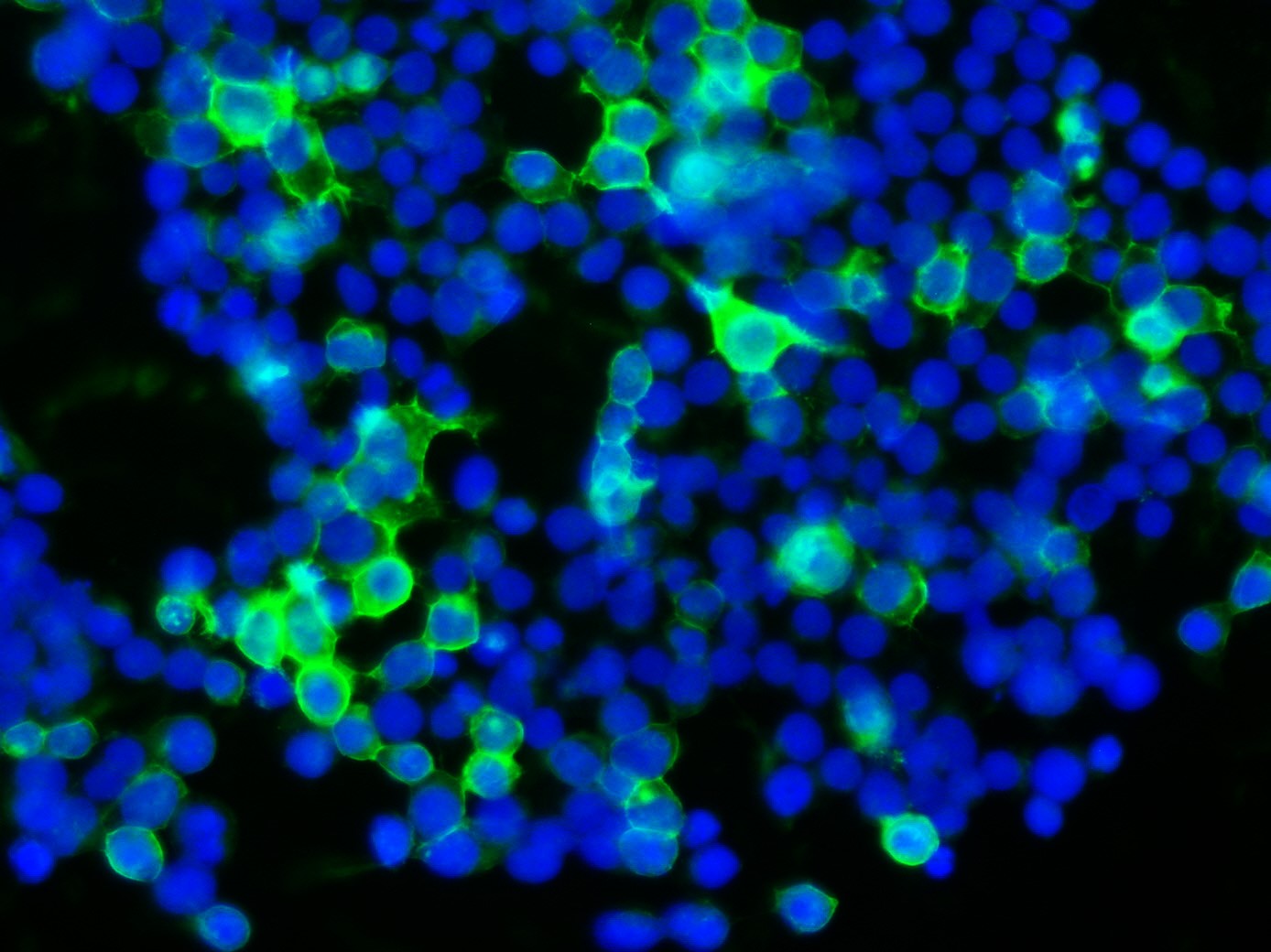

Supplement: S1 Raw Data — (ZIP) [file pntd.0012604.s002.zip › S1_RawData/Raw data/Fig 1/Fig 1E/rAAV-CHIKV-SP/MERGE.JPG]

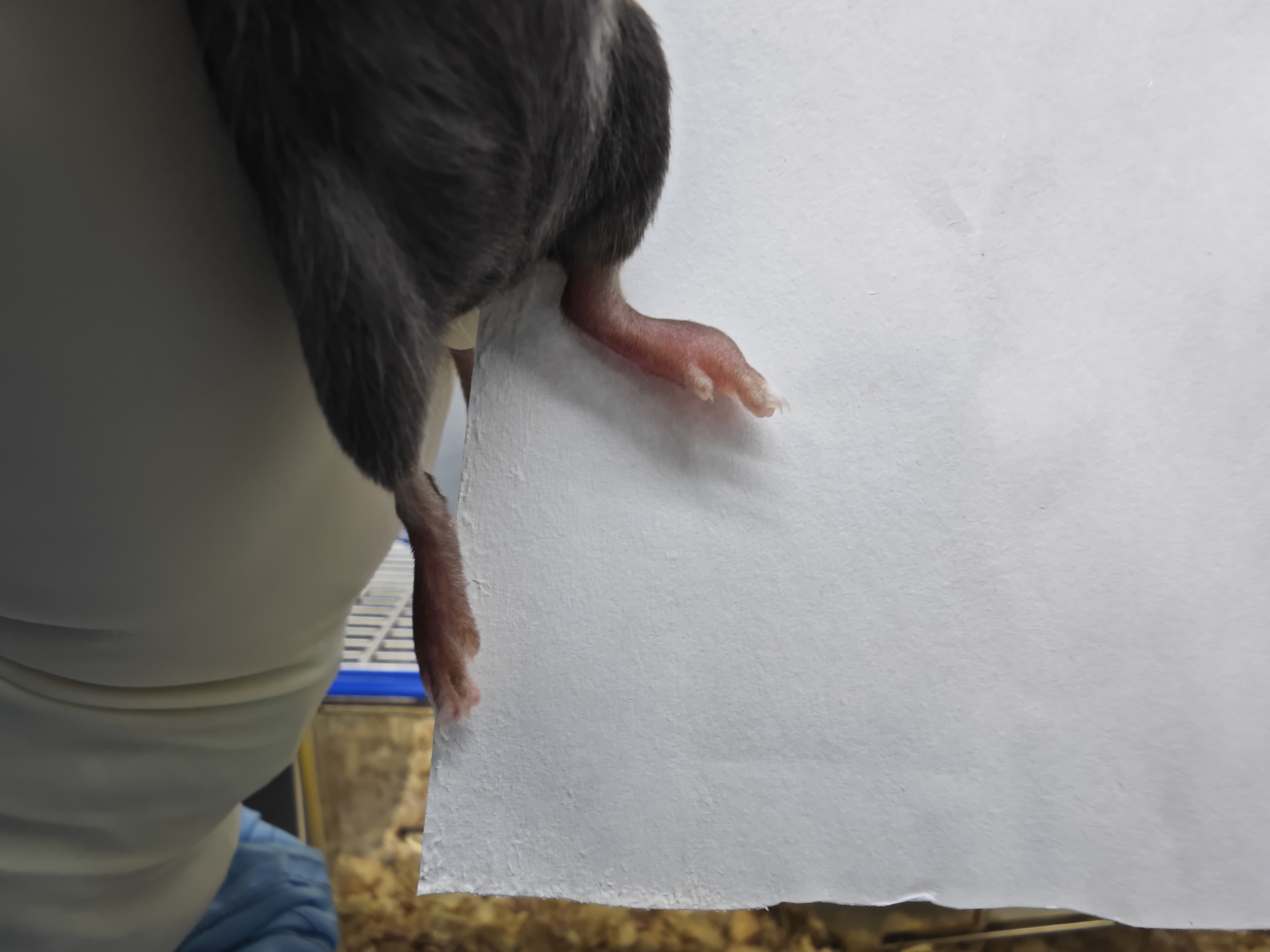

Supplement: S1 Raw Data — (ZIP) [file pntd.0012604.s002.zip › S1_RawData/Raw data/Fig 3/Fig 3C/PBS group.jpg]

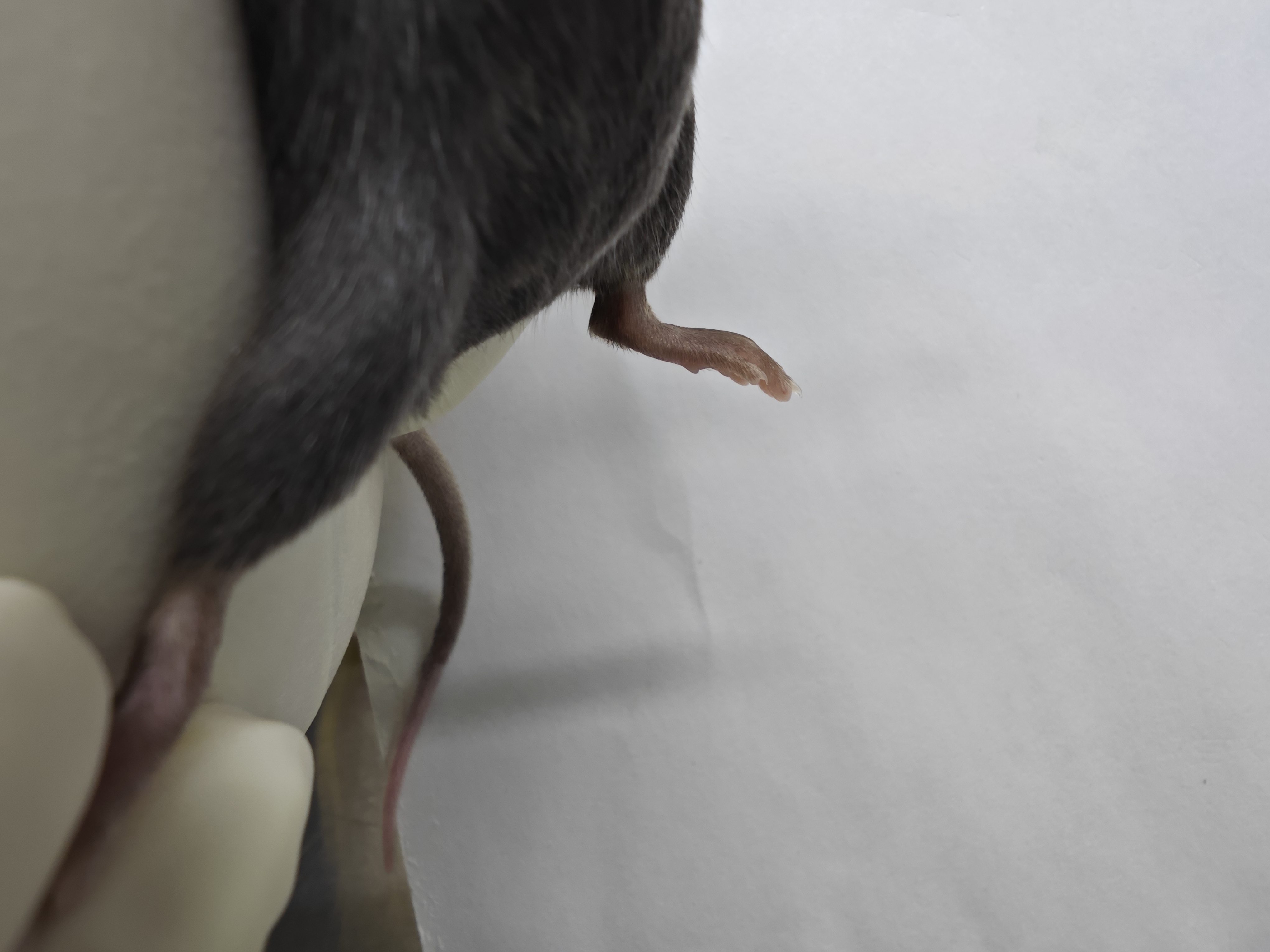

Supplement: S1 Raw Data — (ZIP) [file pntd.0012604.s002.zip › S1_RawData/Raw data/Fig 3/Fig 3C/rAAV-CHIKV-SP group.jpg]

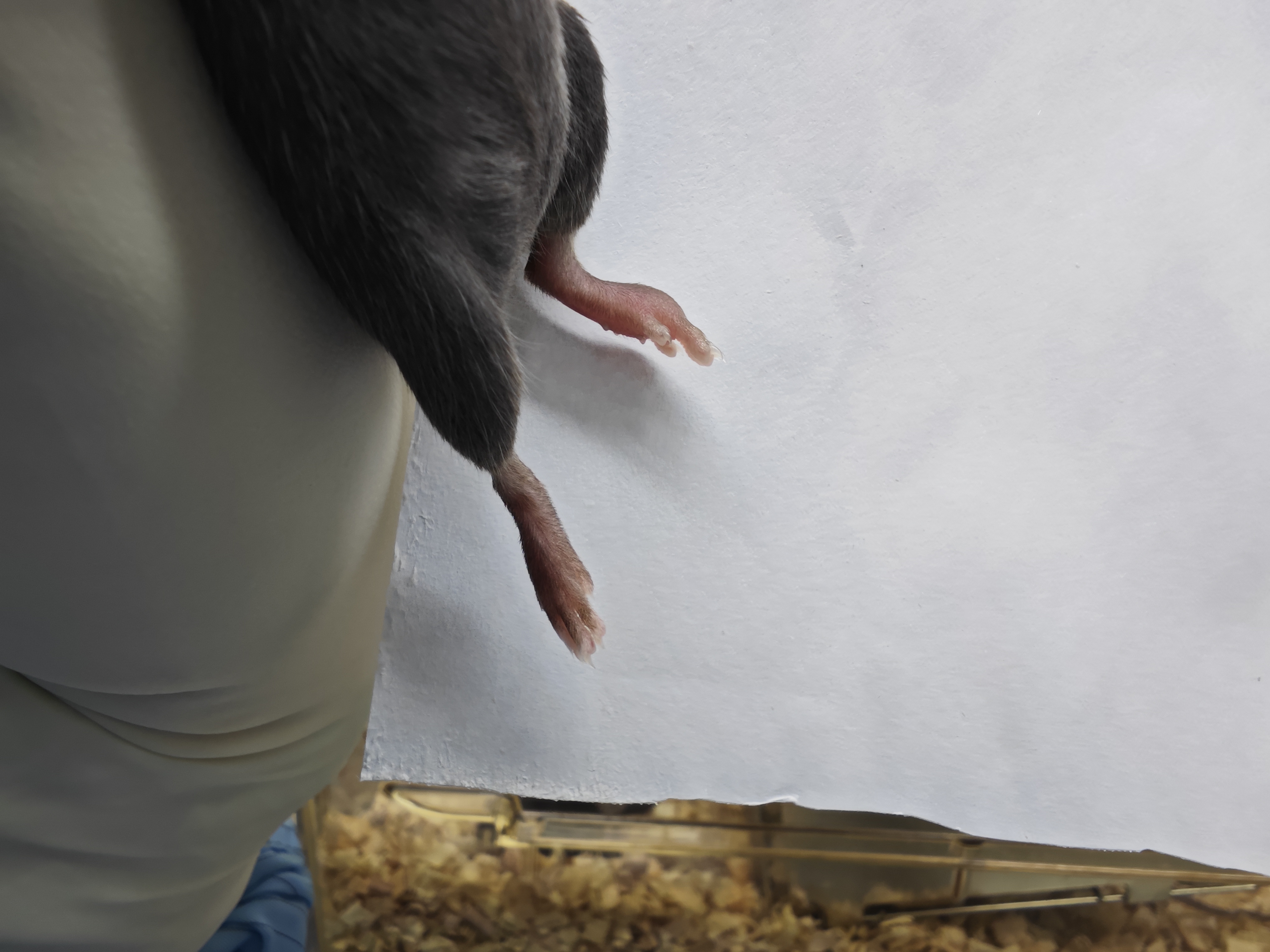

Supplement: S1 Raw Data — (ZIP) [file pntd.0012604.s002.zip › S1_RawData/Raw data/Fig 3/Fig 3C/rAAV-eGFP group.jpg]

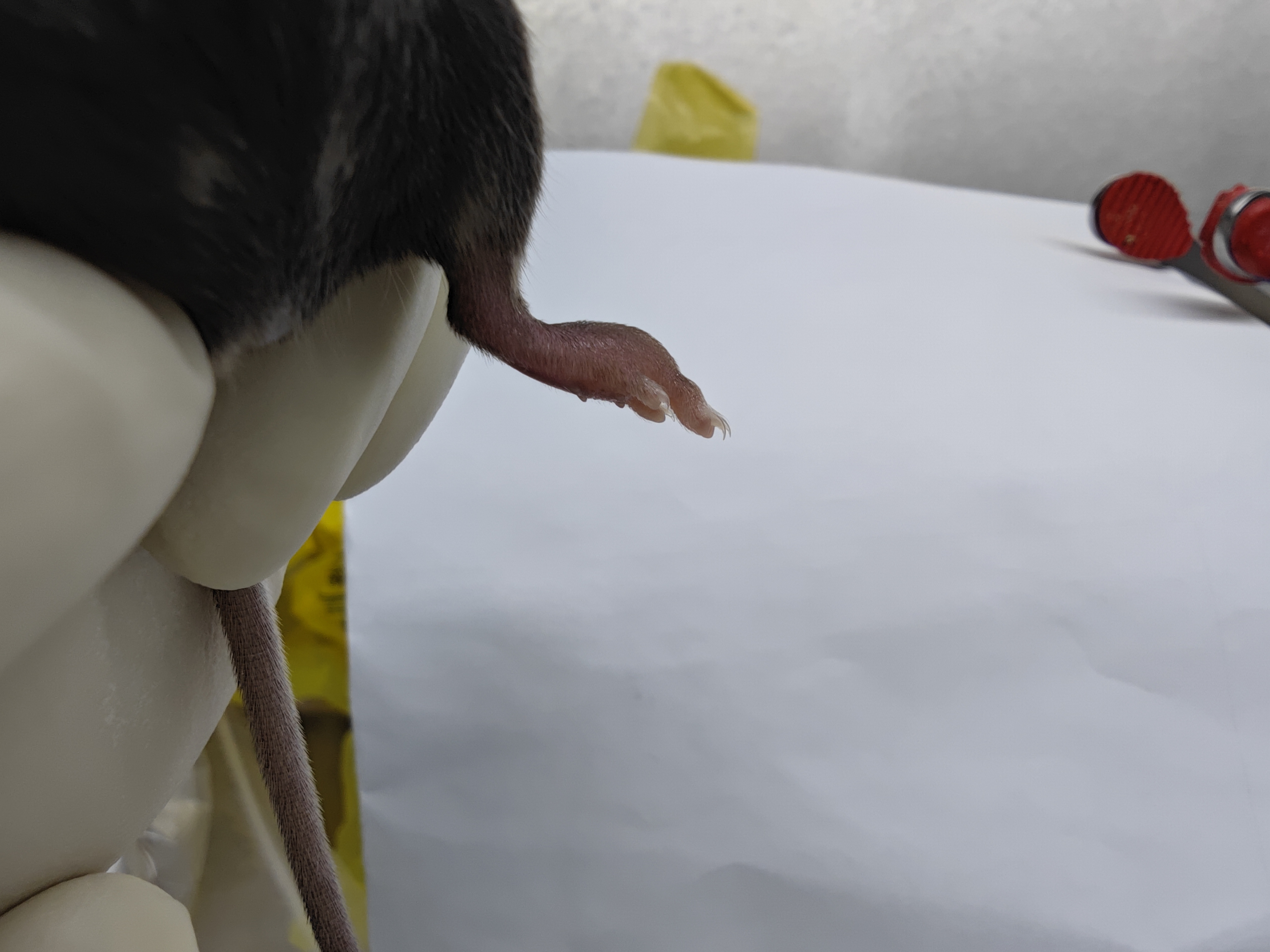

Supplement: S1 Raw Data — (ZIP) [file pntd.0012604.s002.zip › S1_RawData/Raw data/Fig 5/Fig 5C/PBS control.jpg]

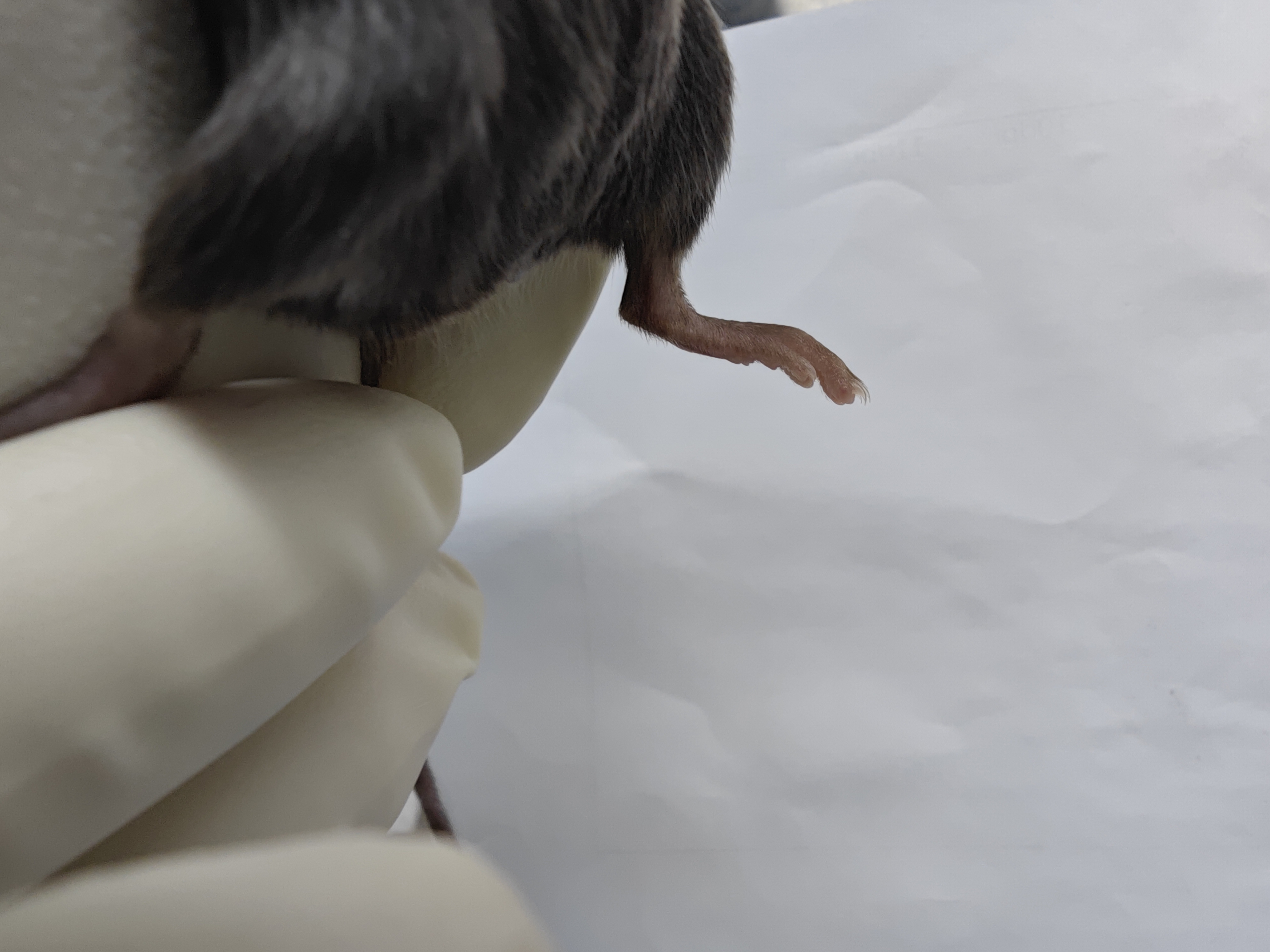

Supplement: S1 Raw Data — (ZIP) [file pntd.0012604.s002.zip › S1_RawData/Raw data/Fig 5/Fig 5C/rAAV-CHIKV-SP-5E10 immunization.jpg]

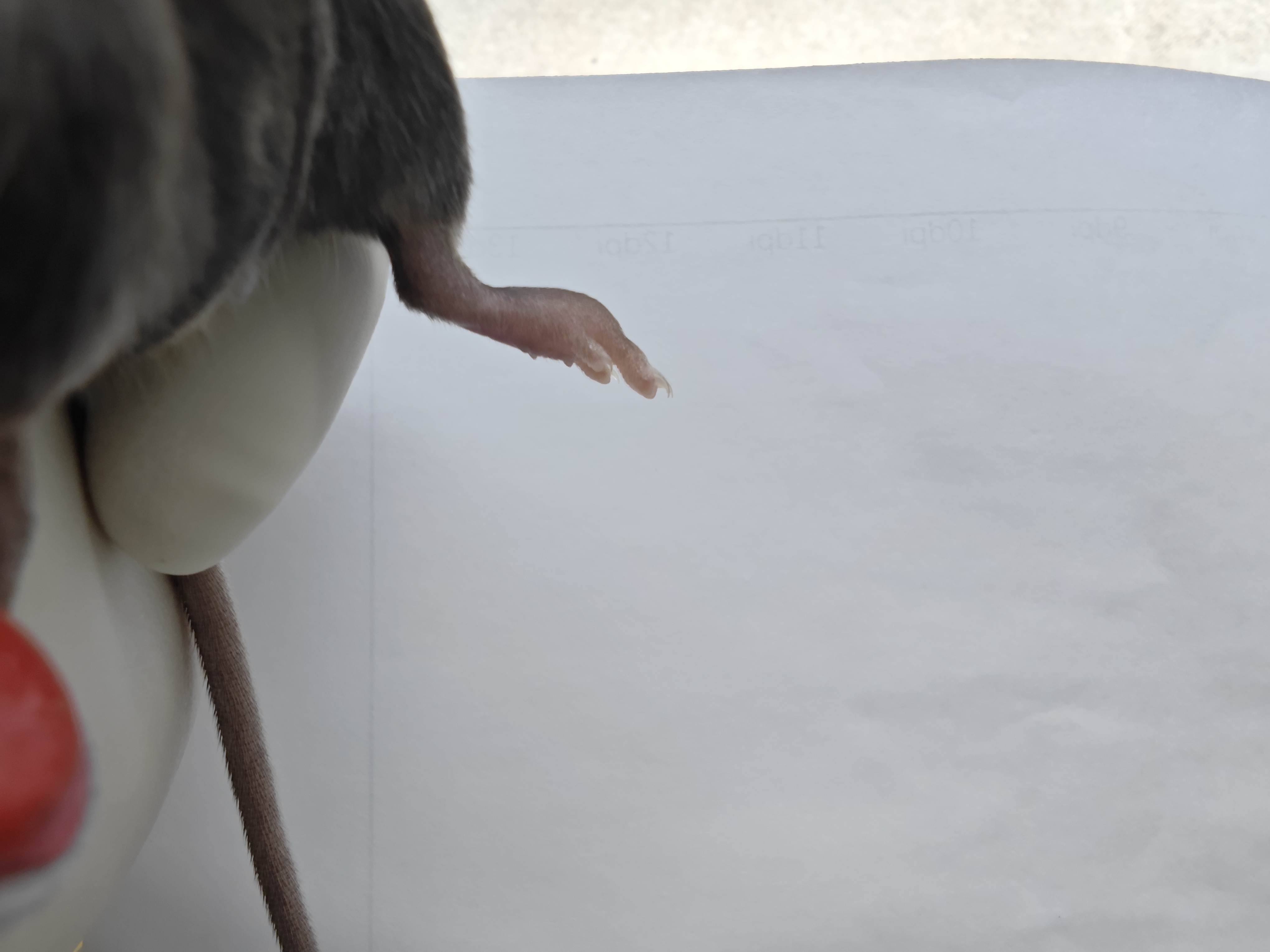

Supplement: S1 Raw Data — (ZIP) [file pntd.0012604.s002.zip › S1_RawData/Raw data/Fig 5/Fig 5C/rAAV-CHIKV-SP-5E8 immunization.jpg]

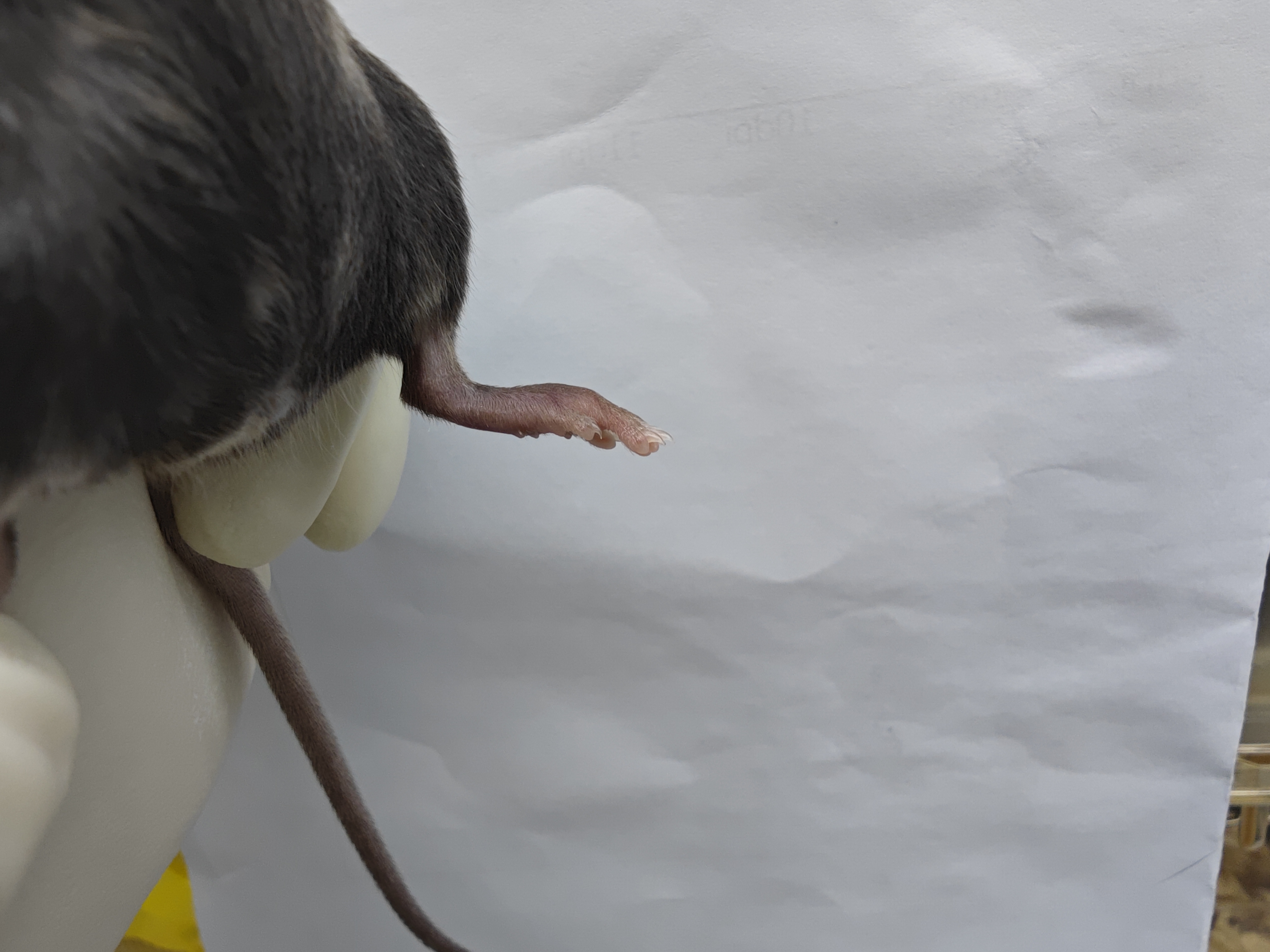

Supplement: S1 Raw Data — (ZIP) [file pntd.0012604.s002.zip › S1_RawData/Raw data/Fig 5/Fig 5C/rAAV-CHIKV-SP-5E9 immunization.jpg]
